# Transcriptomic profiling of host-parasite interactions in the microsporidian *Trachipleistophora hominis*

**DOI:** 10.1186/s12864-015-1989-z

**Published:** 2015-11-21

**Authors:** Andrew K. Watson, Tom A. Williams, Bryony A. P. Williams, Karen A. Moore, Robert P. Hirt, T. Martin Embley

**Affiliations:** Institute for Cell and Molecular Biosciences, Newcastle University, Newcastle upon Tyne, NE2 4HH UK; Biosciences, College of Life and Environmental Sciences, University of Exeter, Devon, UK

**Keywords:** Host-parasite interactions, Microsporidia, Gene expression, Transcriptomics, Evolution

## Abstract

**Background:**

*Trachipleistophora hominis* was isolated from an HIV/AIDS patient and is a member of a highly successful group of obligate intracellular parasites.

**Methods:**

Here we have investigated the evolution of the parasite and the interplay between host and parasite gene expression using transcriptomics of *T. hominis*-infected rabbit kidney cells.

**Results:**

*T. hominis* has about 30 % more genes than small-genome microsporidians. Highly expressed genes include those involved in growth, replication, defence against oxidative stress, and a large fraction of uncharacterised genes. Chaperones are also highly expressed and may buffer the deleterious effects of the large number of non-synonymous mutations observed in essential *T. hominis* genes. Host expression suggests a general cellular shutdown upon infection, but ATP, amino sugar and nucleotide sugar production appear enhanced, potentially providing the parasite with substrates it cannot make itself. Expression divergence of duplicated genes, including transporters used to acquire host metabolites, demonstrates ongoing functional diversification during microsporidian evolution. We identified overlapping transcription at more than 100 loci in the sparse *T. hominis* genome, demonstrating that this feature is not caused by genome compaction. The detection of additional transposons of insect origin strongly suggests that the natural host for *T. hominis* is an insect.

**Conclusions:**

Our results reveal that the evolution of contemporary microsporidian genomes is highly dynamic and innovative. Moreover, highly expressed *T. hominis* genes of unknown function include a cohort that are shared among all microsporidians, indicating that some strongly conserved features of the biology of these enormously successful parasites remain uncharacterised.

**Electronic supplementary material:**

The online version of this article (doi:10.1186/s12864-015-1989-z) contains supplementary material, which is available to authorized users.

## Background

Microsporidia are a group of obligate endoparasitic fungi [1, 2]. They are highly successful pathogens that are able to infect a diverse range of hosts, including species of economic significance, and can also cause disease in immunocompromised humans. Microsporidia were first characterised as the causative agent of pébrine, the disease that contributed to the fall of the European silkworm industry in the 19th century [3]. More recently, the microsporidian *Nosema ceranae* has been implicated in colony collapse disorder affecting bee populations world wide [4]. Documented cases of human microsporidiosis have risen sharply since the onset of the AIDS pandemic, and 14 species of Microsporidia have been described as causing opportunistic infection in immunocompromised humans [5], including *Trachipleistophora hominis,* the focus of the present study, which was isolated from an HIV/AIDS patient in 1996 [6, 7]*.*

Microsporidians are obligate intracellular pathogens, and their genomes have become highly reduced in terms of protein coding content as a result [8–11]. Recent analyses suggest that most gene loss occurred in the common ancestor of microsporidians, leaving a small core of genes that carry out functions that are essential for all eukaryotic cells, embellished by an additional conserved core of genes common to most microsporidians [10, 11]. This novel, Microsporidia-specific core includes genes for a variety of different proteins, many of unknown function, and some of which form expanded gene families in contrast to the general trend of microsporidian genome reduction. Included among the latter are genes for nucleotide transport proteins (NTT) that are present in multiple copies on all but one of the microsporidian genomes sequenced to date [8, 10, 11, 12]. In the absence of ATP production capabilities these proteins, which are among the very few proteins to be functionally characterised for microsporidians, play a key role in the import of ATP and other nucleotides from infected host cells, without which the parasite cannot complete its life cycle [13, 14]. The observation that other proteins, including additional transporters, show similar patterns of retention and expansion [11] suggests that they may also play conserved roles in the microsporidian intracellular lifestyle.

The advent of RNA-Seq provides the opportunity to investigate microsporidian gene expression during the intracellular stages of infection on a genome-wide scale. Gene expression analyses of *Encephalitozoon cuniculi* [15], *Nematocida parisii* [16], *Spraguea lophii* [17], and *Nosema bombycis* [18] have already shown that this technology can be used for microsporidians, highlighting the potential of this technique for studying a group of parasites that cannot be genetically manipulated in the laboratory*.* They have also highlighted strategies by which host cells respond to microsporidian infections, including defence responses mediated by ubiquitation [19], the production of antimicrobial peptides [18], and the perturbation of metabolic pathways [18]. In the present study we have used RNA sequencing to investigate gene expression by *Trachipleistophora hominis* infecting a mammalian (rabbit kidney) cell line, and we compare host expression under infected and non-infected conditions.

Our expression analyses confirm the large coding capacity – 3153 genes – of *T. hominis* refuting a recent suggestion [20] that the large number of genes initially reported might be an artefact of genome annotation. Although our analyses did identify some false gene models, this was balanced by the identification of genes previously missed during the genome annotation, including some that expand the metabolic capacities of *T. hominis* in interesting ways. Parasite gene expression was extremely heterogeneous with 5 % of genes accounting for over 50 % of total gene expression. This includes a strong signature for genes involved in replication and growth, but also a cohort of highly expressed genes that are conserved among microsporidians but are of so far unknown function. We detect strong evidence of functional divergence within gene families including transport proteins that, counter to the prevailing mode of gene loss, have undergone expansion. These data support classical ideas of functional divergence after gene duplication with the most conserved paralogue being the most highly expressed. Analysis of single nucleotide polymorphisms (SNPs) and their allele frequency spectrum strongly suggest that *T. hominis*, like other unikaryotic Microsporidia [16, 21, 22], is diploid and raises the possibility of a sexual stage in its lifecycle. The detection of active insect-derived transposons suggests that *T. hominis* – which is an opportunistic pathogen of humans – has an insect host in nature. Transcriptional profiling of the host is consistent with a generalised cellular shutdown upon infection with *T. hominis*, with the upregulation of host pathways for ATP production, amino sugar and nucleotide sugar metabolism: these pathways potentially complement gaps in parasite biosynthesis predicted by *in silico* analyses.

## Results and discussion

### Reproducibility of host-parasite transcriptomics

**Fig. 1:**
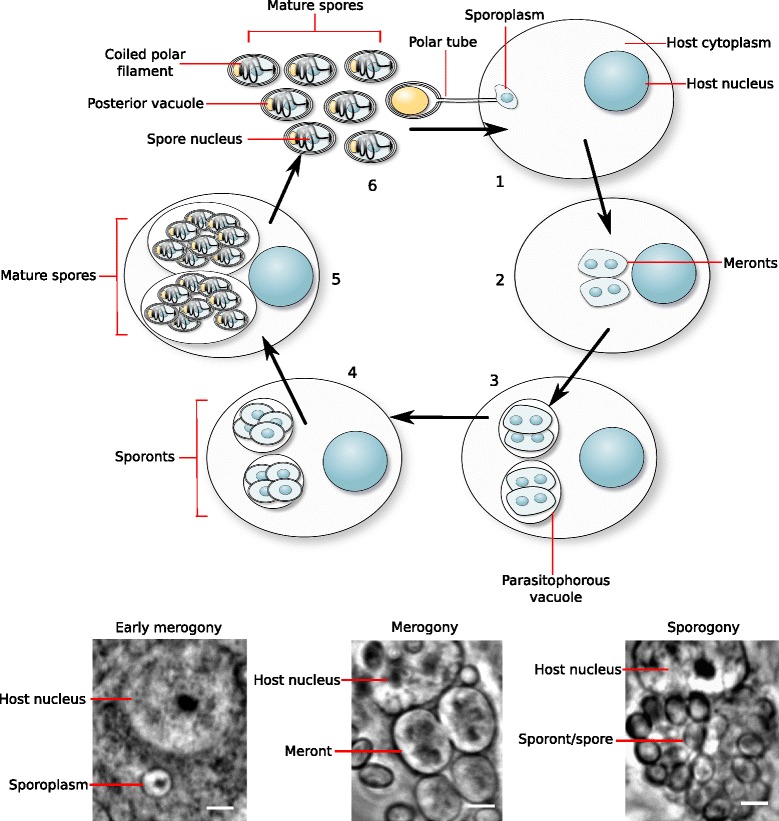
A schematic lifecycle of *Trachipleistophora hominis* with phase contrast images of representative intracellular stages*.* In stage 1, the *T. hominis* spore injects its contents into the host cell via a proteinaceous polar tube. This uninucleate sporoplasm is the earliest stage of infection of the host cell [119]. Following injection, the parasite – now called the meront - grows and begins to proliferate in the host cell (merogony, the second stage of the parasite lifecycle) [6, 7]. It is unknown how many cycles of proliferation meronts undergo in the host cell before stage three of the lifecycle begins, the transition from meronts to spore-forming sporonts. In Microsporidia this is marked by the deposition of an electron-dense material on the surface of the parasite, and in *T. hominis* may represent the timing of formation of the parasitophorous vacuole, an additional membrane surrounding the parasite within the host cell [6, 7, 119]. During spore formation (sporogony) the microsporidian spore wall is assembled, and *T. hominis* undergoes a further round of proliferation [6, 7]. Eventually mature spores form inside the host cell (stage 5). These spores may either eject their polar tubes from within their host cell and infect neighbouring cells, or else be released from the cell in stage 6 of the parasite lifecycle, allowing dissemination of the infection to new cells or new organisms [119]

**Fig. 2:**
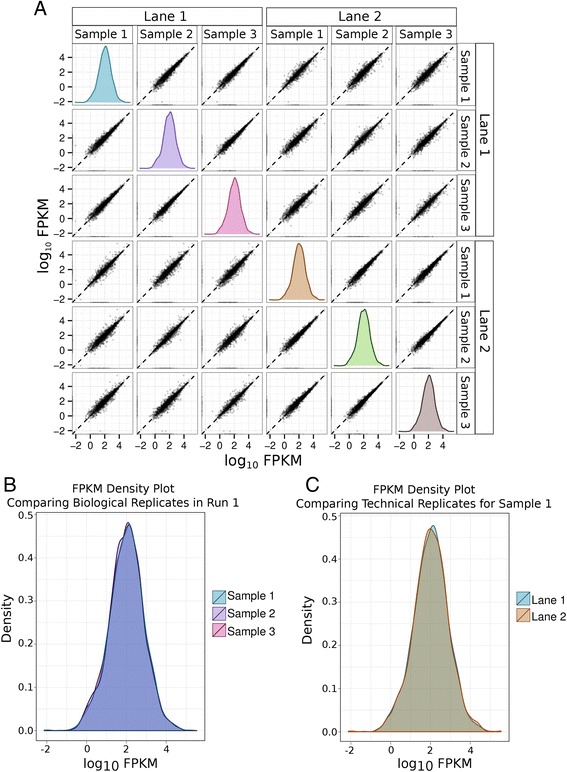
Robustness and reproducibility of transcriptomic analysis for *T. hominis*. In this figure, each sample is an individual biological replicate of *T. hominis* infected RK-13 cells from which total RNA was purified. All of these samples were sequenced on two different lanes of the Illumina sequencing chip to provide technical replication. The strong positive correlation of transcript abundance, as measured by Fragments Per Kilobase per Million mapped reads (FPKM) between replicates in all cases is indicative of high levels of reproducibility between both biological and technical replicates. **a** Pairwise comparisons of *T. hominis* transcript log_10_ FPKM values between biological replicates (samples) and technical replicates (sequencing lanes); density plots generated in cummeRbund [109] represent the distribution of log_10_ FPKM values for that replicate. **b** FPKM density plot overlay comparing the distribution of *T. hominis* transcript FPKM values between individual biological replicates (samples) from a single sequencing lane. **c** FPKM density plot overlay comparing the distribution of *T. hominis* transcript FPKM values between technical replicates (different sequencing lanes) from a single sample (Sample 1). The levels of reproducibility between replicates for the expression of host genes were equally very high, this data is summarised in Additional file 1, Figure S1

*T. hominis* is an obligate intracellular parasite grown in laboratory co-culture within rabbit kidney (RK) cells [6]. We harvested total RNA from three biological replicates of infected RK cells seven days post inoculation, at which point ~60 % of RK cells in each flask were infected with *T. hominis*. At this stage the community of *T. hominis* cells was a mixture of different life cycle stages including thick walled spores and pre-spore stages (sporonts and sporoblasts) as well as the intracellular sporoplasm (newly geminated parasite inside the host cell) and replicative or meront stage (Fig. 1). Although we used a bead beating method similar to one previously shown [10] to lyse *T. hominis* spores in our RNA extractions, the resistant nature of the spore-forming stages of the parasite lifecycle may make lysis less efficient, which could lead to an enrichment of transcripts from replicative stages in the total RNA pool; possible implications of this bias are discussed in more detail below. In parallel we also isolated total RNA from three biological replicates of uninfected RK cells, in order to compare patterns of host expression under the two conditions. We obtained 2.3 × 10^7^ sequencing reads from the infected cells, with 7.7 % of these reads mapping to the *T. hominis* genome. The reproducibility between biological and technical replicates was very high, both for pairwise comparisons of the expression of individual genes between replicates and the overall distribution of expression levels across all transcripts (Fig. 2 (*T. hominis*); Additional file 1: Figure S1 (rabbit)). These results indicate that our analysis of the *T. hominis* and host transcriptomes was highly reproducible; thus, the potential biological implications can be explored in a meaningful way.

### *T. hominis* has more genes than small-genome microsporidians

The *T. hominis* genome (~11.6 Mbp haploid genome size) was initially reported to contain 3266 predicted open reading frames (ORFs), which is over 1000 more genes than the ~2000 open reading frames predicted for the best studied small genome (~2.3–2.5 Mbp haploid genome size) species of *Encephalitozoon* [20]. It has been suggested that the predicted number of *T. hominis* genes may be an overestimate due to over-prediction of small genes [20]. Indeed, some ORFs (113) appear to be unique to *T. hominis*, with no homologues identified in its closest sequenced relative *Vavraia culicis* [23, 24], or any other microsporidian [10, 11]. Additionally, *T. hominis* encodes a large family of novel leucine rich repeat proteins (117 ORFs) that includes many fragmented ORFs, indicative of ongoing pseudogenisation [10]. It is therefore possible that the estimated coding capacity of *T. hominis* includes some false positives, particularly given the highly derived nature of most microsporidian gene sequences [10, 20]. We obtained evidence for the expression of 2958 (90 %) of the 3266 annotated *T. hominis* ORFs (Additional file 2: Table S1), including 85 % of the leucine-rich repeat genes, suggesting that most are genuine ORFs. However, we detected expression for fewer (73 %: 83 of 113) *T. hominis*-specific genes, consistent with the idea that some of these are false-positive calls. Balancing this reduction in the predicted gene count based on transcription, we obtained evidence for an additional 292 transcripts that were not predicted as ORFs in the original genome project. One hundred and fifty-five (80 %) of these transcripts are located within regions of ambiguous genomic sequence or near the ends of scaffolds; the difficulty in annotating these regions may explain their absence from the original *T. hominis* genome annotation [10].

Ninety seven of the 292 novel transcripts lacked an ORF suggesting that they might be *T. hominis* noncoding RNAs, although they did not give significant matches to the noncoding RNAs already included in the Rfam database [25, 26]. They also appear to be missing from other microsporidian genomes as searched using BLASTN. Despite this, one of the transcripts, XLOC_000764, was in the upper 95th percentile of overall expression levels; that is, its expression level was higher than 95 % of detected transcripts, suggesting that it plays a physiologically relevant role. The remaining 195 transcripts are predicted to contain ORFs of which 89 had significant hits to the nr protein database at a BLASTX E-value cutoff of 0.01. The two most highly expressed of the 89 shared significant similarity to partial ORFs (VCUG_01670 and VCUG_016701) annotated in the *Vavraia culicis* genome [24], the closest sequenced relative of *T. hominis* [23]. The putative *T. hominis* protein is 564 amino acids in length and contains a series of 12 tandem glycine-asparagine repeats. A search using HHPred [27, 28] suggests that these are similar to a class of repeats present in over 30 % of *Plasmodium falciparum* genes. Their function in *Plasmodium falciparum* is unknown, but it has been suggested that they may interact with host proteins [29]. Consistent with this idea, the *T. hominis* gene has an N-terminal signal peptide [30], suggesting that it might be secreted or localised on the surface of the parasite.

**Fig. 3:**
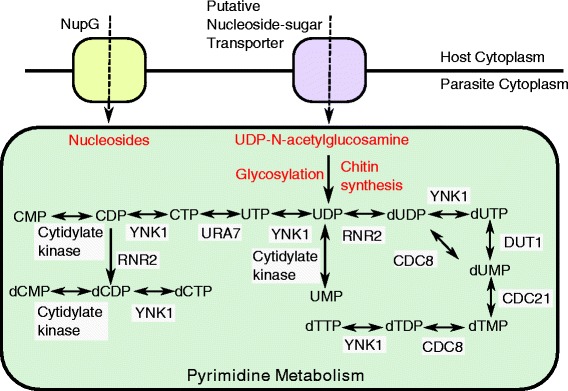
A potential route for pyrimidine and chitin precursor acquisition by *T. hominis. De novo* routes for pyrimidine biosynthesis are not present in Microsporidia, but scavenger pathways allow conversion between different pyrimidines [11]. The import of only one pyrimidine from the host cell would, in principle, enable the biosynthesis of other pyrimidines via scavenger pathways, because interconversions between bases are possible. NupG-like transport proteins have been hypothesised to transport nucleosides and thus act as a source of pyrimidine precursors [16], but the specificity and subcellular localisation of these genes are currently unknown. A putative UDP-N-acetyl glucosamine transporter provides another potential source of pyrimidines in *T. hominis* and *V. culicis*: the imported metabolite can feed into chitin synthase and glycosylation pathways, both of which may liberate free pyrimidine

**Fig. 4:**
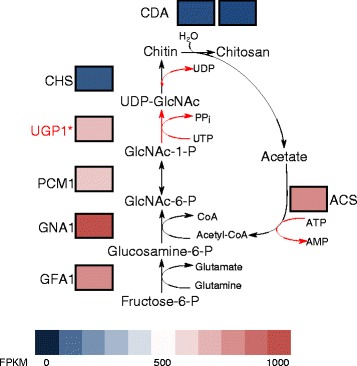
A drive for UDP-N-acetylglucosamine biosynthesis with low-level spore wall production. The heatmap shows the levels of expression of genes within the chitin biosynthesis pathway, with blue representing low levels of expression and red as high. GFA1, glutamine-fructose-6-phosphate aminotransferase; GNA1, glucosamine-6-phosphate N-acetyltransferase 1; UGP1, UDP-N-acetylglucosamine pyrophosphorylase; PCM, phosphoacetylglucosamine mutase; CHS, chitin synthase; CDA, chitin deacetylase; ACS, acetyl-CoA synthetase. The significantly lower expression levels of chitin synthases compared to the rest of the pathway leading to UDP-N-acetylglucosamine production suggest that chitin synthesis is not very active in this sample, consistent with the low expression of other enzymes involved in spore wall formation

Twenty-five of the newly identified transcripts show significant similarity to genes outside of the microsporidian clade, including several broadly-distributed eukaryotic genes previously thought to be absent from the genome of *T. hominis*. These include exportin, a component of the nuclear export machinery, as well as homologues of deoxyhypusine hydroxylase [31], the Rea1 AAA-ATPase [32], and the 60S ribosomal protein L29 [33]. We found transcript evidence for three new transport proteins, including an amino acid/auxin permease of the AAAP family, a putative cation transporting P-type ATPase, and a member of the DMT superfamily of drug and metabolite transporters that includes a UAA transporter family domain (pfam:08449) associated with UDP-N-acetyl-glucosamine:UMP antiporter activity. Published data demonstrate that microsporidians can import purine nucleotides using nucleotide transport (NTT) proteins [11, 13, 14] but as yet there is no evidence for the transport of pyrimidine nucleotides by these transporters. Based upon *in silico* predictions it appears that microsporidians cannot make pyrimidines *de novo* so there is a transport gap that needs to be filled [11, 13, 14]. UDP-N-acetylglucosamine is the direct monomeric precursor for chitin synthesis, an integral component of the microsporidian spore wall [34], but it is also biosynthesised by the mammalian host cell in which it plays roles as a co-enzyme, signalling molecule, and precursor for glycosylation [35, 36]. The pyrimidine UDP is liberated from UDP-N-acetylglucosamine during chitin polymerisation by chitin synthase and during glycosylation, so as well as providing chitin precursors this novel transporter could potentially provide the starting substrate to make pyrimidines needed for *T. hominis* DNA and RNA biosynthesis (Fig. 3). Genes within the chitin biosynthesis pathway in *T. hominis* are generally expressed at similar levels in our analysis (600–900 fpkm), however expression of the terminal components of the pathway, chitinase and chitin synthases, were much lower (20–30fpkm) (Fig. 4). These observations suggest that in the proliferative stages of the microsporidian lifecycle, UDP-N-acetylglucosamine is either being used primarily for UDP-liberating glycosylation reactions, or being accumulated for chitin production during sporogony. We also obtained transcriptomic evidence for a previously unannotated *T. hominis* homologue of a UDP-N-acetylglucosamine pyrophosphorylase, an enzyme also encoded on the genomes of *Vavraia*, *Encephalitozoon*, *Anncalia*, *Edhazardia* and *Vittaforma.* This enzyme catalyses the conversion of UTP and N-acetyl-alpha-D-glucosamine 1-phosphate to UDP-N-acetylglucosamine and diphosphate. This means that, in addition to potential acquisition of UDP-N-acetylglucosamine from the host, *T.* hominis encodes a complete pathway for its biosynthesis (Fig. 4). The potential importance of this enzyme for spore wall formation in Microsporidia makes it a potential target for therapeutic intervention. Thus, recent studies have shown that UDP-N-acetylglucosamine pyrophosphorylase is essential for the survival of *Trypanosoma brucei* in its bloodform lifecycle stage [37], and chemicals that can selectively inhibit the *Trypanosoma brucei* UDP-N-acetyl pyrophosphorylase have been identified [38].

In conclusion, the transcript data from our experiments appear to be very reproducible for both host and *T. hominis*. The data validate the majority of gene models originally predicted from the *T. hominis* genome sequence and identify some previously missed genes, providing evidence for a total of 3153 transcribed genes and making the *T. hominis* gene complement one of the largest identified for microsporidians so far [20].

### Overlapping transcription in a gene-sparse microsporidian genome

We detected 140 *T. hominis* transcripts encoding more than one ORF, of which 113 overlap on the genome assembly. The remaining 27 do not overlap on the genome, but the intergenic region between them was spanned by RNA-Seq reads. This suggests that overlapping transcription occurs in *T. hominis,* as previously reported for the small genome species *E. cuniculi* and *A. locustae,* where a similar number of multi-ORF transcripts (144) was identified [39, 40]. Interestingly, our data suggests that overlapping transcription is not necessarily linked [40, 41] with genome compaction, because gene density in *T. hominis* is actually lower than for yeast [10]. One possibility is that overlapping transcription provides a mechanism for co-regulating the expression of particular genes [42]. If so, then this mode of regulation is poorly conserved over evolutionary time, because transcriptional overlap is conserved for only one gene pair across *E. cuniculi*, *A. locustae*, and *T. hominis* [40]: ribosomal protein L6 (THOM_0162) and RNA polymerase III transcription factor IIIC subunit 5 (THOM_0163), which overlap in the 3′ UTR.

### Low levels of splicing in *T. hominis*

A total of 85 introns were predicted in *T. hominis* based upon the presence of conserved *E. cuniculi* intron motifs [10]. Surprisingly, we obtained transcriptomic evidence for the splicing of only a single gene – 40S ribosomal protein S23 – at this conserved intron motif (JUNC00000002 in Additional file 3: Table S2); this gene was spliced efficiently, with 93 % of detected transcripts being spliced, higher than the splicing efficiency detected for any spliced gene in the *E. cuniculi* transcriptome [15]. This gene was also one of only two genes for which splicing was detected in transcripts from the microsporidian *Spraguea lophii*, and the intron has a similar length [17]. Splicing in general in Microsporidia appears to be inefficient for short introns [17], with splicing rates of less than 15 % identified for several *E. cuniculi* transcripts [15]. This potentially explains our inability to detect splicing at the great majority of characterised intron motifs in the *T. hominis* transcriptome. By contrast, we obtained evidence for 13 introns where RNA sequencing reads suggest a spliced exon boundary. One of these novel junctions (JUNC00000048 in Additional file 3: Table S2) is found in the third most highly expressed transcript in our dataset, and its presence is supported by a greater number of reads than the originally predicted 40S ribosomal protein S23. Surprisingly, the remaining new experimentally identified introns lack the classical intron motif previously described in *E. cuniculi* [43]*,* and an alignment revealed no unique novel motif common to the group (Additional file 4: Figure S2).

### Highly expressed *T. hominis* genes

The top 5 % (170 genes) of genes accounted for over half (58 %) of all detected transcription in *T. hominis* (Additional file 5: Figure S3)*.* Fifty three percent of these highly transcribed ORFs belong to a core conserved set of microsporidian genes defined by Nakjang et al. [11] as those encoded by 9 of the analysed 11 sequenced microsporidian genomes. Of these highly expressed core genes 32 % encode rRNA or ribosomal proteins, and 31 % encode other essential elements of eukaryotic cell biology including cytoskeletal proteins, transcription and translation factors, cell division proteins (e.g., NudC), histones, and molecular chaprones. Similar functional gene groups (E.g. Ribosome biogenesis and protein translation factors) were significantly enriched in highly expressed genes during proliferative growth of fission yeast (*Schizosaccharomyces pombe*) [44], suggesting the pattern of expression observed in *T. hominis* may reflect the signatures of its rapid growth and replication in the host cell. The highly expressed core microsporidian genes also include a large number of microsporidian hypothetical proteins of unknown function (37 %). In fission yeast, groups of functionally related genes tend to be expressed at similar levels [44], suggesting that if the same pattern applies to Microsporidia these uncharacterised proteins may also play important and as yet unknown roles in core parasite biology and proliferation.

The inclusion of molecular chaperones of the HSP40/DNAJ and HSP70 families and protein disulfide isomerase among highly expressed core *T. hominis* genes is intriguing. High expression of HSP70 has been identified in several different microsporidian clades [41], suggesting it is a common feature of the group. Chaperones have been identified as important virulence factors in bacterial and eukaryotic pathogens, including intracellular bacteria [45]. Published work has demonstrated that intracellular bacteria and bacteria cultured under conditions that introduce a population bottleneck often over-express chaperones to maintain functionality under an increased mutational load [46–50]. The high levels of chaperonin expression in several microsporidians*,* which also experience bottlenecks during transmission and show high rates of sequence evolution [6, 10], suggest that these intracellular eukaryotes are behaving in the same way [41].

Replication and biosynthesis are energy-requiring processes and hence *T. hominis* must either make or acquire ATP and GTP during proliferation. Genome analyses suggest that *T. hominis* has a complete glycolytic pathway [10] and we detected expression of all of the relevant enzymes in the transcriptome, but only glyceraldehyde 3-phosphate dehydrogenase was in the top 5 % of transcripts. The most highly expressed glycolytic enzymes are PBA, GAPDH and PGK, with GAPDH and PGK respectively providing NAD^+^ reduction and ATP synthesis. These data suggest that *T. hominis* is potentially making some of the ATP it needs, but the cell stage where this occurs is not resolved by our data. Quantitative immuno-localisation of PGK protein, the first ATP-generating step of glycolysis, suggests that the protein is mainly, but not exclusively, inside *T. hominis* spores rather than vegetative cells [10]. It has also been previously suggested that glycolysis occurs mainly in the spores of another microsporidian, *Paranosema grylli* [51]. Interestingly, transcripts for the most abundant protein identified in spores, Polar Tube Protein 3, were detected at only modest levels in the RNA sequencing data, with an average of 18.1 FPKM ±12.3 SD (within the 30th percentile for expressed genes in the dataset). Indeed, no genes known to be associated with spore wall formation (spore wall proteins, polar tube proteins and chitin synthases) were found in the top 5 % of highly transcribed genes. One possibility is that the lysis procedure used to extract total RNA may not lyse *T. hominis* sporonts, sporoblasts and spores very efficiently; if so, then the extracted RNA would be enriched for transcripts from replicative stages of the parasite lifecycle despite the mixed infection. Alternatively, these more quiescent stages of the parasite lifecycle might show an overall reduction in levels of gene expression, as observed in the nonreplicating stages of the fission yeast cell cycle [44], so that they will naturally be represented at lower levels in the total RNA pool Conversely, high levels of expression were observed for a number of genes involved in DNA replication and proliferation, consistent with an enrichment of transcripts from the actively proliferating stages of the parasite, and suggesting that some ATP production by glycolysis may occur in these stages of the parasite lifecycle.

Few metabolic enzymes appeared in the top 5 % of expressed transcripts, consistent with genomic predictions that *T. hominis* must import many of the substrates it needs for biosynthesis directly from the infected host cell [10, 11]. One highly expressed enzyme is nucleotide diphosphate kinase (YNK) [52], which was also highly abundant in proteomic analyses of highly purified spores [10], YNK is predicted to play a key role in supporting *T. hominis* intracellular proliferation by converting nucleotides or nucleoside diphosphates to their triphosphate forms, the precursors for both RNA and DNA synthesis and sources of cellular energy. High levels of expression were also observed for dUTPase, another enzyme predicted to be involved in nucleotide biosynthesis [53]. The most highly transcribed metabolic enzyme was an asparagine synthetase A (asnA, THOM_2136, InterPro ID: IPR004618). Among Microsporidia, coding sequences for this protein are found on the genomes of *T. hominis, V. culicis, Enterocytozoon bienieusi, N. ceranae* and *Nosema pernyi*, and were likely acquired by lateral gene transfer from bacteria [10, 11]. Among eukaryotes, AsnA is almost exclusively found in parasites [10] including *Typanosoma brucei* and *Leishmania donovani*, where it is essential for survival [54, 55]. In bacteria, AsnA is responsible for the reversible transamination of aspartate to asparagine in the presence of ATP and ammonia. *Leishmania and Trypanosoma* AsnA, the only characterised eukaryotic homologues, have a broader specificity, and are able to use glutamine as a nitrogen donor to generate glutamate [55, 56]. One possibility is that microsporidian AsnA may play important roles in interconversion between essential amino acids, including glutamine, an important precursor to both chitin biosynthesis for spore wall formation and glutathione (GSH) biosynthesis required in parasite detoxification systems. This ORF contains an aminoacyl-tRNA synthetase (class II) domain (InterPro ID: IPR004364) suggesting that it may also function to add asparagine to its cognate tRNA. Its specificity to parasitic eukaryotes, coupled with its functional importance, high expression level, and the availability of an AsnA crystal structure make the protein a promising potential drug target [55].

Maintaining supplies of glutamine is important for the generation of GSH, a detoxifying molecule required for the prevention of damage by reactive oxygen species [57]. The *T. hominis* detoxification system also includes thioredoxin reductases, peroxidases, glutathione reductases and superoxide dismutase [10]. While all identified components of this pathway are expressed in *T. hominis*, the highest expressed in our dataset, and only component in the 95th percentile of overall expression levels, was iron/manganese superoxide dismutase (SOD), which reduces and detoxifies superoxide molecules (O_2_^−^) [10]. The largest biological source of superoxide species is as a by-product in the production of ATP by oxidative phosphorylation, which *T. hominis* does not carry out but which we show is upregulated in host cells during infection by *T. hominis* (see below). *T. hominis* SOD may protect against oxidative stress generated by the host cell as it supports both its own survival and parasite proliferation. Previous work [58–60] has indicated that oxidative stress in the host cell is elevated during infection; therefore, a robust detoxification system is likely to be important for parasite survival, as observed in some bacterial infections [61].

One metabolic pathway known to be essential and that has been functionally characterised in Microsporidia is iron-sulphur cluster biogenesis [62]. Iron-sulphur clusters are required for the activity of key proteins needed for microsporidian replication, including DNA polymerase. The metabolic pathway for the biogenesis of iron-sulphur clusters is compartmentalised, starting in the microsporidian mitosome (remnant mitochondrion) and ending in the cytosol [62]. This allows us to examine the variability in levels of transcript abundance between different compartments in a single linked pathway that should be required throughout the parasite lifecycle. The only highly expressed gene in the pathway (in the top 5 % of expressed transcripts) was Dre2, a cytosolic component of iron-sulphur cluster biogenesis [63]. Interestingly, Dre2 also plays a role in inhibiting free radical-induced apoptosis [63]; given that other detoxifying enzymes are also highly expressed in *T. hominis*, it may be this function of Dre2 that drives its high expression level. Consistent with this idea, the other components of iron-sulphur cluster biogenesis are expressed at significantly lower and similar levels, suggesting that genes in the same pathway may be generally expressed at similar levels in *T. hominis*, as observed in fission yeast [44].

Surface-located transport proteins are predicted by genome analyses to be fundamental for supporting the replication of *T. hominis* and other microsporidians by importing substrates from infected host cells [3, 10, 11, 13, 14]. The expression of *T. hominis* proteins related to known transporters, or annotated as potential transporters, is very heterogeneous within structural types. Only three predicted transport proteins are found in the top 5 % (>2210 FKPM) of expressed genes and these do not include any of the *T. hominis* nucleotide (NTT1-4) transporters for which functional data is available [14]. The most highly expressed of these – NTT4 – appears in the 92^nd^ percentile of expression levels. NTT4 is one of four paralogous nucleotide transporters that are expressed on the surface of replicating parasites [14], where they function to transport purine nucleotides including ATP and GTP, for energy and/or biosynthesis [14]. Of the three highly expressed transporters, the first and third in terms of expression are hypothetical transporters of unknown specificity while the second is a putative inorganic phosphate transport protein. Given the potential importance of the NTT transporters it seems possible that these more highly transcribed transporters also support important, albeit currently uncharacterised, cellular functions.

The most highly transcribed of the three membrane proteins (THOM_1886) appears to be specific to *T. hominis* and its closest sequenced relative, *V. culicis,* as determined by sensitive PSI-BLAST [64] and HMMER [65] based searches. The THOM_1886 protein is predicted to include 7 transmembrane domains and was annotated as a putative transport protein [10]. Its expression level is more than 1500x that of the average transporter in our study (22209 FPKM ± 5483 SD) suggesting that, in addition to the conserved common core of microsporidian genes, lineage-specific innovation is also important for parasite biology.

### Levels of *T. hominis* gene expression are correlated with gene history and conservation among microsporidians

**Fig. 5:**
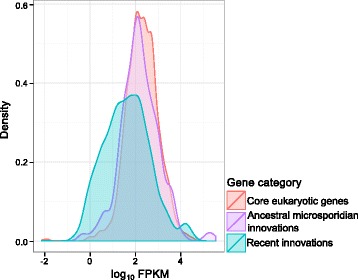
Levels of *T. hominis* gene expression are correlated with gene history and conservation among microsporidians. *T. hominis* genes can be classified according to the period in evolutionary history when they first arose: core eukaryotic genes, shared with most or all other eukaryotes and encoding fundamental features of eukaryotic cell biology; ancestral microsporidian innovations, found only in *T. hominis* and other microsporidians; and recent innovations, genes found only in *T. hominis* and its close relative *V. culicis.* This density plot shows the distribution of expression levels (as log_10_FPKM) for *T. hominis* genes in each of these three categories. Mixed-effects modelling indicates that core eukaryotic genes and ancestral microsporidian innovations are expressed at significantly higher levels than recently-evolved *T. hominis* genes (*P* = 0), but that there is no significant difference in expression patterns between the former two classes (*P* = 0.166); the recently-evolved genes also show a much broader range of expression than the older gene classes, perhaps reflecting greater variation in functional constraints within this group

Comparative analyses of the genome of *T. hominis* with other microsporidian genomes have demonstrated that genome evolution has been a dynamic process, in which the loss of ancestral gene families has been partially offset by the gain of new microsporidia-specific genes [8–11, 16, 17, 66]. In this broader evolutionary context, *T. hominis* genes can be classified into three major groups: core eukaryotic genes – that is, core microsporidian genes defined in Nakjang at al. [11] that were also found in most or all eukaryotes; ancestral microsporidian innovations, or core microsporidian genes that evolved in the common ancestor of all microsporidia (and thus are not identified in other eukaryotes); and recent innovations (for example THOM_1886) that are only found in *T. hominis*, or that are shared between *T. hominis* and its close relative *V. culicis.* To evaluate the relationship between evolutionary conservation and expression level in *T. hominis*, we compared the expression levels of the genes in these three classes using a linear mixed-effects model (Fig. 5).

Our analysis indicated that core eukaryotic genes and ancestral microsporidian innovations were both expressed at significantly higher levels than recently-evolved genes specific to the *T. hominis*/*V. culicis* lineage (*P* = 0), but that there was no significant difference in expression levels between the two more highly-expressed classes (*P* = 0.166). The consistently high expression levels for core eukaryotic genes are not, in themselves, particularly surprising: this category includes genes involved in basic cellular processes such as DNA replication and repair, mitochondrial iron-sulphur cluster assembly, intracellular trafficking and in some metabolic pathways such as glycolysis and the pentose phosphate pathway. However, the equally high level of expression observed for ancestral microsporidian innovations is interesting because it implies that genes which first evolved in the common ancestor of microsporidia, and which were then conserved across the group, are as important to microsporidians – by the measure of transcript abundance - as genes encoding the fundamental eukaryotic cellular componentry.

By contrast, genes specific to the *T. hominis*/*V. culicis* lineage are expressed at significantly lower levels than other genes in the parasite (*P* = 0); we focused on genes shared between these close relatives to minimise the impact of false ORF calls on our analyses. Most of these genes are expressed (735 out of 862, or 85 %), but not necessarily at high levels; as can be seen from Fig. 5, this class of recently evolved genes displays a broad range of expression levels. The more heterogeneous distribution of expression levels for recently evolved genes is consistent with a recently proposed model [67] for the gradual emergence of proto-genes from previously non-coding sequence. Under this model, some new, fortuitously expressed genes acquire important functions and are maintained by selection, while others do not and will eventually be lost to drift and pseudogenisation. It is possible that this process of genomic innovation underpins recently evolved host-parasite interactions for these two species, both of which are thought to infect insects as their natural hosts [10, 68]. Consistent with this hypothesis, the *T. hominis/V. culicis*-specific families are enriched for signal peptides (*P* = 1.2 x 10^−10^, Fisher’s exact test) [11], suggesting that the proteins in these families may be localised to the parasite cell surface, part of the infective polar tube, or secreted into the host cell.

### Expression divergence in expanded *T. hominis* gene families

In contrast to the general trend of reductive evolution among microsporidians, a number of *T. hominis* gene families have expanded through gene duplication. Gene duplication is important in the evolution of gene family function, because duplication events can relax selective constraints allowing the functions of one or both paralogues to change [69, 70]. Consistent with a role for duplication and functional divergence in microsporidian evolution, Nakjang et al. [11] and Heinz et al. [14] found evidence of sequence divergence at conserved amino acid residues following microsporidia-specific duplications in the Hsp90 chaperone, Ste24 metalloprotease, NTT nucleotide transporter, ZiP zinc ion permease, SulP sulphate permease and NupG nucleoside transporter families. Intriguingly, members of these expanded gene families tend to be expressed at above-average levels in *T. hominis* (*P* = 3 x 10^−4^, linear mixed-effects model).

**Fig. 6:**
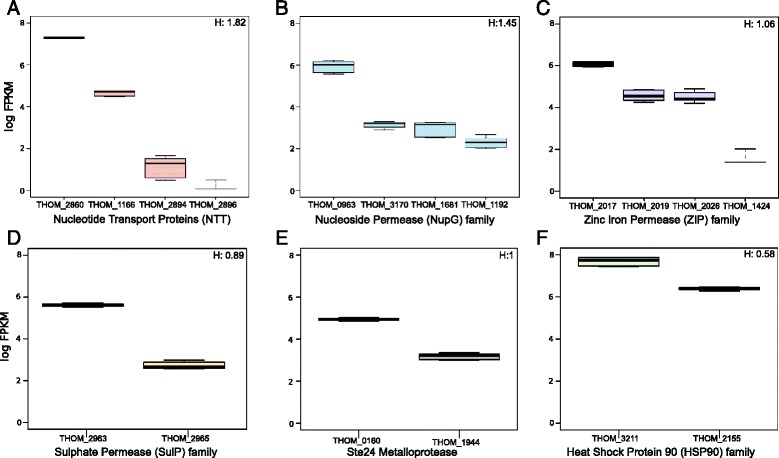
Members of gene families with evidence of sequence divergence also show variable levels of gene expression. Expression levels (log_10_ FPKM) for the members of gene families in which functional divergence was previously detected at the sequence level [11]. In each case, the most highly expressed paralogue is also the most highly conserved based on single gene trees. H, the heterogeneity index calculated for each gene family, is also shown (see Materials and Methods). **a** Nucleotide transport proteins (NTTs). **b** NupG-related nucleoside permeases. **c** Zinc iron permase (Zip) family. **d** Sulphate permease (SulP) family. **e** Ste24 metalloprotease family. **f** Heat shock protein 90 (HSP90) family

Figure 6 summarises expression levels for the functionally characterised *T. hominis* nucleotide (NTT) transport proteins [14] and *T. hominis* members of the microsporidian gene families investigated by Nakjang et al. [11]. The variation in expression level is clearly correlated with the evolutionary history of the gene family: in all of these cases, the most highly conserved family member, in terms of conservation of critical residues or branch length in gene family trees [11, 14], is also the most highly expressed. These data are consistent with a model of functional divergence whereby one conserved, highly-expressed paralogue continues to carry out the ancestral function, while other duplicates experiencing reduced selective constraint can gain new functions [71–73]. Any new functions, which could include stage-specific expression, different cellular location or substrate affinity or specificity, will need to be identified through experiment. For example, proteomics data already suggest that NTT4, the most highly expressed member of the gene family (Fig. 6), is the main NTT transporter located within the *T. hominis* spore [10]. In *Encephalitozoon cuniculi* the NTT transporter family has undergone an independent expansion [14]. This expansion was followed by divergence in both sequence and localisation, with one family member (EcNTT3) localised to the mitosome while the other three are located on the surface of replicating parasites [13]. The correlation between sequence divergence and transcript abundance we observe in *T. hominis* is maintained in *E. cuniculi* [15], with divergent family members (EcNTT3 and EcNTT4) expressed at lower levels (mean FPKM 139 for EcNTT3, 96 for EcNTT4) than the more highly conserved family members (EcNTT1 and EcNTT2 – 402 and 977 FPKM respectively).

To evaluate variation in expression among microsporidian gene duplicates more systematically, we analysed expression for all duplicate families identified by Nakjang et al. [11] containing at least two paralogues in *T. hominis*. We calculated the standard deviation of FPKM values within each family, and normalised by the per-family mean (see Materials and Methods) (Additional file 6: Table S3). Expanded *T. hominis* families were then ranked by this metric to identify the families showing the most extreme expression divergence. Plotting these scores revealed an inflection point in the distribution of the metric, above which we considered within-family expression to be highly heterogeneous (Additional file 7: Figure S4). The most heterogeneous family identified by this approach included a family of retrotransposon-encoded reverse transcriptases; some family members had no detectable expression, suggesting ongoing pseudogenization as might be expected for transposable elements. The group of *T. hominis* families showing the greatest expression divergence also includes the hexokinase gene family, whose paralogues in the microsporidian *N. parisii* have been suggested to manipulate host metabolism following secretion into the host cell [16]. *T. hominis* encodes four hexokinases of which two include predicted signal peptides [11], consistent with the hypothesis that they may be secreted into the host cell. One of the remaining copies (XLOC_001491) is the most highly expressed member of the family, again consistent with the idea that the most highly conserved member of a duplicated family continues to perform the ancestral function.

### Parallel horizontal transfers of transposons implicate an insect host in the lifecycle of *T. hominis*

*T. hominis* is one of several microsporidians that retain elements of the RNA interference machinery [10]. The core components of this machinery, Dicer and Argonaute, are both expressed by *T. hominis* during infection but are not in the top 5 % of expression. The RNAi machinery is hypothesised to play a role in defence against transposon activity in Microsporidia [10]. Consistent with this hypothesis, we obtained evidence for the expression of 58 of the 110 annotated transposons in the *T. hominis* genome. Combined with evidence for transcription of transposons in *Edhazardia aedis* [41], these data suggest that active transposons pose an ongoing threat to genome integrity in microsporidians more generally.

**Fig. 7:**
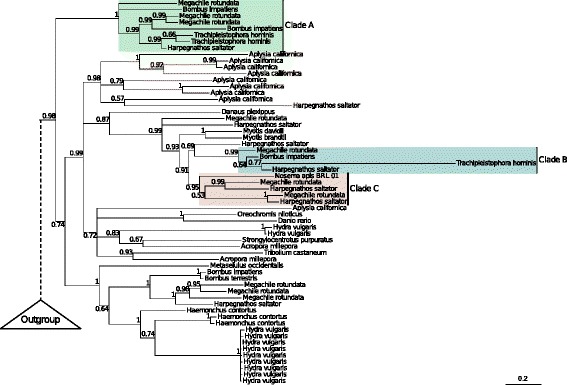
Phylogenetic analysis of PiggyBac transposons suggests a natural insect host for *T. hominis*. A Bayesian phylogeny of *T. hominis* PiggyBac transposases inferred under the C20 model [117] in PhyloBayes [118]. Support values are Bayesian posterior probabilities, and branch lengths are proportional to the expected number of substitutions per site. The tree topology supports two recent, independent transfers of PiggyBac elements from hymenopteran insects into *T. hominis.* In both cases, the ant *Harpegnathos saltator* is recovered as the closest relative of the *T. hominis* sequence, although with variable posterior support. We also identify a transfer from insects into the microsporidian *Nosema apis*, a honeybee parasite. Clade A – An insect clade including two *T. hominis* PiggyBac elements, as identified in [10]. Clade B – A distinct clade of insect elements, including the newly discovered *T. hominis* PiggyBac element. Clade C – Clade including *Nosema apis* and insect PiggyBac elements

Although *T. hominis* is an opportunistic parasite of immunocompromised humans, its natural host remains unknown. *T. hominis* can proliferate within artificially infected mosquitoes under experimental conditions, but these infections have not been observed in nature [74]. One of the novel transcripts identified in this study showed similarity to a PiggyBac transposase [75]. This transcript maps to a previously unannotated portion of the *T. hominis* genome, and is therefore distinct from the PiggyBac element reported in the original genome annotation [10]. The best BLAST hits to the novel PiggyBac element include transposable elements from insects but not other Microsporidia, raising the possibility that *T. hominis* gained the element in a recent horizontal transfer that occurred after the divergence of *T. hominis* from its close relative *V. culicis*. A PiggyBac element identified as recently acquired in bats has been demonstrated to retain activity when inserted into both human and yeast cells, highlighting the capacity of this particular family of transposons for inter-species transfer [76]. Phylogenetic analysis of the novel *T. hominis* element strongly suggests that it was recently acquired from an insect, and probably a member of the Hymenoptera (ants, bees and wasps; Fig. 7 and Additional file 8: Figure S5). The *T. hominis* sequence forms a strongly supported clade with PiggyBac elements from bees (*Bombus impatiens* and *Megachile rotundata*) and the ant *Harpegnathos saltator* (Fig. 7, Clade B). Interestingly, this transfer appears to have occurred independently of the previously identified PiggyBac acquisition from insects in *T. hominis*, which branches in a separate insect clade with maximal posterior support (1.0 posterior probability; Fig. 7, Clade A). In both cases, the most closely related sequence is from Jerdon’s jumping ant (*Harpegnathos saltator*), although the posterior support for this relationship is variable (0.99 in Clade A, and 0.77 in Clade B). The phylogeny of the two separate *T. hominis* PiggyBac elements provides consistent support for the hypothesis that *T. hominis* infections of humans may represent opportunistic zoonoses from a natural hymenopteran host. Interestingly, we also identified a separate novel horizontal transfer from insects into the microsporidian *Nosema apis*, a honeybee parasite [77], providing further support for the horizontal transfer of host-derived transposable elements into Microsporidia (Fig. 7, Clade C) [78–80].

### Patterns of single nucleotide polymorphism reveal that *T. hominis* is diploid

Microsporidia replicate inside their host cell, and can only exist outside it as a resistant and infectious spore [3]. This would be a barrier to sex between two different parasite cells, which would require multiple independent infections of a single host cell. Nonetheless, limited evidence for a sexual reproduction cycle has been identified in several microsporidians [81]. The early morphological characterisation of *Ambylospora* identified a meiosis-like stage of division including karyogamy, the fusion of two haploid nuclei to form a single diploid nucleus [82]. Recent studies of the *Nosema/Vairimorpha* lineage have also provided some evidence for sex and recombination [81]. Although proliferating *T. hominis* cells can contain multiple nuclei, nuclear fusion has never been observed [6, 7]. Our RNA-Seq data presents an opportunity to investigate the ploidy of *T. hominis* and to test whether sexual reproduction may be possible.

**Fig. 8:**
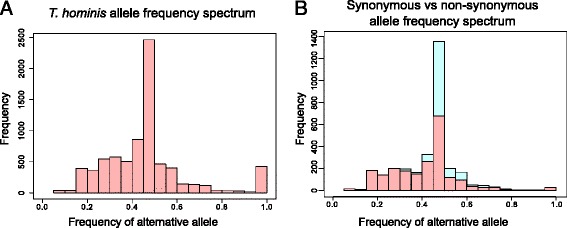
The allele frequency spectrum of SNPs in expressed *T. hominis* transcripts suggests a diploid phase to the parasite lifecycle. **a** The allele frequency spectrum (reference and alternative alleles) for *T. hominis* SNPs. The clear peak at an allele frequency of 0.5 suggests that *T. hominis* is diploid. **b** Allele frequency spectra with synonymous and non-synonymous SNPs plotted separately. The distributions are similar, providing no evidence for reduced expression of non-synonymous alleles

We identified 7596 variant sites (polymorphisms) in the *T. hominis* transcriptome, with a total of 7654 possible variants. These included 7120 single nucleotide polymorphisms (SNPs), 314 insertions and 220 deletions. Plotting the allele frequency spectrum of the variations reveals a clear peak at a frequency of 0.5 (50 % reference genome allele, 50 % alternative allele) (Fig. 8a). Our *T. hominis* populations are likely to be clonal, both because their obligate intracellular lifecycle results in a population bottleneck in each generation, and also because our experimental isolate has been passaged repeatedly in cell culture. In addition, population-level variation would not be expected to give rise to a peak at 0.5, unless two distinct populations had somehow been maintained in a 50:50 ratio. Given these considerations, the simplest interpretation of the observed allele frequency spectrum is that the genome of *T. hominis* is diploid in at least some stage of its lifecycle and that, at least in the majority of cases, both alleles are expressed. *T. hominis* is unikaryotic – that is, it has one nucleus per spore – and so our results are consistent with analyses suggesting that other unikaryotic microsporidians are also diploid [16, 21, 22]. The diploidy of *T. hominis* and other unikaryotic Microsporidia supports the notion that diplokaryotic Microsporidia are likely to be tetraploid [83], containing two diploid nuclei as observed in the diplomonad *Giardia lamblia* [84]. The diploidy of *T. hominis* raises the possibility that it occasionally has sex, although the density of SNPs in our dataset was not sufficiently high to evaluate the possibility of recombination or linkage disequilibrium. Although the *T. hominis* spore is unikaryotic, the intracellular stages of its lifecycle divide by a combination of binary division and plasmotomy, the division of a single cell producing multinucleate progeny [6, 7]. This raises the possibility that meiosis could still be triggered in multinuclear intracellular stages of the parasite lifecycle.

A total of 5496 SNPs were identified within annotated *T. hominis* ORFs, with 2175 non-synonymous and 2933 synonymous changes. SNPs were identified in 1551 ORFs in total, with 1071 of these ORFs including non-synonymous changes, leading to possible protein variants. Although we cannot exclude the possibility that some non-synonymous mutations might be beneficial, we expect the majority to be deleterious, particularly given the frequent genetic bottlenecks experienced by our artificially maintained experimental population. 388 changes were more severe in nature, either including frameshifts or alterations to start and stop codons. In principle, the deleterious effects of these mutations could be suppressed in a diploid organism by preferential expression of the reference allele. However, the peak at 0.5 is still observed in the frequency spectrum for non-synonymous alleles (Fig. 8b), implying that synonymous, non-synonymous and reference alleles are all expressed at similar levels. 34 % of all SNPs occurred in “core” microsporidian genes – those shared among at least 9 of 11 sequenced Microsporidia and predicted to play an important role in the parasite. The expression of these variants is perhaps surprising due to their potential impact on protein function. However, it is important to remember that this study examines a single population of *T. hominis,* and that many of these SNPs may be en route to elimination by negative selection. Another possibility is that the high levels of expression we observed for key molecular chaperones may help to suppress the phenotypic effect of these deleterious mutations, as has previously been reported for intracellular bacteria [46].

### Response of the RK-13 cell line to infection with *T. hominis*

Recent studies have begun to shed light on the mechanisms by which microsporidians exploit their hosts [13, 14, 85, 86], but we still know almost nothing about the host response to infection at the molecular level. We quantified host gene expression to identify genes and pathways that were differentially expressed in RK-13 cells during infection with *T. hominis* compared to uninfected cells (Additional file 9: Table S4). These analyses provide a first snapshot of the impact of *T. hominis* infection on the host cell transcriptome. We identified 1734 transcripts that showed significant changes in expression in the RK-13 cell line during infection with *T. hominis* (Additional file 10: Table S5). KEGG categories [87] were assigned to these transcripts using the KOBAS annotation pipeline [88]; nine KEGG categories were enriched for differentially expressed transcripts (Additional file 11: Table S6); that is, these categories contained more genes whose expression levels changed in response to infection than would be expected by chance, allowing us to explore the general effects of infection on the host cell.

Our analysis suggests that the host experiences a generalised cellular shutdown in response to *T. hominis* infection, with the down-regulation of the great majority of genes involved in the KEGG pathways for the cell cycle, meiosis, DNA replication, and ribosome biogenesis (Additional file 12: Figure S6). Several other host cell pathways contained a mixture of both up-regulated and down-regulated genes relative to the uninfected control; these pathways are clearly disrupted during *T. hominis* infection, but the overall effect on the host is difficult to predict from changes in transcript abundance. The pathways included those involved in focal adhesion, extracellular matrix-receptor interactions, renal cell carcinoma, oxidative phosphorylation, and pyrimidine biosynthesis. Although these patterns are complex and difficult to interpret, they were also consistent across our three biological replicates, and therefore represent reproducible perturbations of host cell pathways.

The only host cell pathways in which the majority of changes were up-regulations relative to the control were amino sugar and nucleotide sugar metabolism (Additional file 12: Figure S6), potentially leading to increased production of nucleotide sugars by the host. Similar changes to silkworm metabolism were observed during infection with *Nosema bombycis*, suggesting that this may be common feature of microsporidian infection [18]. Candidate nucleotide sugar importers have been identified in *T. hominis* [10] and other Microsporidia, consistent with the idea that microsporidians might manipulate host metabolism to increase production of required substrates. One potential mechanism for the manipulation of host metabolism that has already been proposed is the secretion of hexokinase into the host cell [16]. As in mammalian cancer cells [89], Microsporidia infected RK-13 cells had a modulated hexokinase expression profile compared to healthy cells, possibly to the benefit of the intracellular parasite. The two hexokinase isozymes (HKI and HKII) with the highest affinity for glucose in mammals [90] were significantly differentially expressed in the host during infection, with an increase in HKI and a decrease in HKII. HKI is believed to have a primarily catabolic function, driving glycolysis and ATP production [90] – an essential molecule for *T. hominis* growth and replication. These changes in gene expression draw striking parallels to other host-parasite systems, where complex changes in host energy metabolism [91, 92] and pyrimidine biosynthesis [93] are associated with infection.

A number of pathway regulators were also differentially expressed in RK-13 cells during *T. hominis* infection, providing insights into the potential mechanisms that might underpin some of the observed changes in transcript levels of metabolic genes. We observed significant up-regulation of host 5′-AMP-activated protein kinase catalytic subunit alpha-2 (PRKAA2) and peroxisome proliferator-activated receptor gamma co-activator 1-alpha (PPARGC1A) during *T. hominis* infection, both of which are reported to promote energy metabolism and mitochondrial biogenesis [94, 95]. PRKAA2 is additionally implicated in shutting down ATP-consuming pathways including cell proliferation [94], consistent with our observation of decreased expression of genes in this pathway.

Linked to the above inference of increased host ATP production coupled with reduced consumption, we also observed a significant decrease in the expression of host pyruvate dehydrogenase lipoamide kinase isozyme 4 (PDK4) during infection. This kinase represses metabolism through the phosphorylation and inactivation of pyruvate dehydrogenase, the enzyme that converts pyruvate to acetyl-coA, thereby linking glycolysis and the citric acid cycle [96]. Decreased expression of PDK4 would lead to increased activity of pyruvate dehydrogenase, promoting citric acid cycle-based metabolism and, under normal oxygen conditions, increased ATP production [96, 97]. The increased glucose required to support the elevated metabolic demand might be acquired by increased import since the glucose transporter GLUT9 [98] is highly up-regulated transporter during infection by *T. hominis*.

Changes in expression in the ubiquitination pathway have been recently implicated in the immune response of *C. elegans* to *N. parisii* infection [19], raising the possibility that the ubiquitin system may be part of a common host response to microsporidian infection. Although the expression levels of several host ubiquitination genes were altered upon *T. hominis* infection, the pathway as a whole was not enriched in differentially expressed genes in our analysis, suggesting that this does not represent a major component of the host cell response to infection by *T. hominis*.

## Conclusions

Our transcriptomics data was highly reproducible for parasite and host, and confirmed that *T. hominis*, with ~3150 genes, has one of the largest coding capacities among microsporidians [10, 20]. Although our data rejected some of the shortest predicted gene models, this was compensated by the identification of genes that were previously missed by the genome annotation. Some of these, including transporters that may acquire pyrimidines and enzymes that function in chitin biosynthesis, may plug what were previously considered to be gaps in the metabolic capacity of the parasite.

Gene expression for the parasite was highly biased towards growth and replication, consistent with published microscopic data [6, 7] demonstrating rapid parasite proliferation after infection. Intriguingly, a proportion of the highly expressed transcripts are encoded by conserved microsporidian genes of unknown function, suggesting there is much still to discover about the core biology of these highly successful parasites. Expression within expanded gene families, including key transport proteins, was highly variable. In most cases the most highly conserved members of gene families were also the most highly expressed, consistent with evolutionary models in which duplication can free individual paralogues to diversify in function while preserving the ancestral function in the conserved copy. The expression of genes confined to *T. hominis* and its close relative *Vavraia culicus* was also more heterogeneous than was observed for core genes. Some of this lineage-specific innovation was highly expressed – in particular a membrane protein of unknown function - but much of it was not. This class of genes is also enriched for signal peptides [11] suggesting that some may be secreted or exposed on the surface of the parasite where they can interact with host targets. Our results contribute to a growing body of work supporting the idea that the evolution of contemporary microsporidian genomes is highly dynamic and innovative, and that while the initial transition to intracellular parasitism catalysed a drastic reduction in genome size and coding capacity shared by all microsporidians, important lineage-specific differences continue to evolve.

Our data strongly suggest that *T. hominis* is diploid and demonstrate the presence of a large number of non-synonymous SNPs, many of which are expected to be deleterious, that are equally distributed between alleles. Many of these SNPs may eventually be eliminated by negative selection but, as already suggested for intracellular bacteria [46–49], the high levels of chaperonin expression that we observed may also suppress the phenotypic effects of these deleterious mutations in Microsporidia [41]. It has been demonstrated that artificially infected mosquitoes can support the replication of *T. hominis* [74], but the natural host of this opportunistic pathogen of humans is currently unknown. We identified transcripts from a novel PiggyBac element that, together with a previously identified element of insect origin [10], strongly suggest that the natural host for *T. hominis* belongs to the hymenoptera.

The response of eukaryotic host cells to microsporidian infection is only just beginning to be investigated. Our data, which were highly reproducible between biological and technical replicates, suggest a generalized cellular shutdown by infected cells compared to uninfected rabbit kidney cells. Several other host pathways displayed a reproducible mixture of up-regulated and down-regulated genes relative to the uninfected control; these pathways are clearly disrupted but the overall effect on the host and its relationship to the activities of the parasite are difficult to predict based solely upon these data. We did observe an up-regulation of host amino and nucleotide sugar metabolism: this has also been reported for silkworms infected with *Nosema bombycis*. These are among substrates predicted to be imported by microsporidians to plug gaps in their reduced metabolism [11, 16], so it is possible that these changes are to the benefit of the parasites. There is some evidence that host ATP production might be increased in combination with reduced host energy consumption. This could potentially benefit a parasite that is dependent on the host cell for most of its ATP and purine nucleotides for DNA and RNA biosynthesis [14].

## Methods

### Culture of *T. hominis* infected RK-13 cells

*T. hominis* was grown in co-culture with RK-13 cells [6] grown in minimal essential medium (MEM) containing 10 % heat inactivated foetal calf serum and antibiotics (Penicillin/Streptomycin (0.1 mg/ml), Ampicillin B (1 μg/ml) and Kanamycin sulphate (0.1 mg/ml)). A single 175 cm^2^ flask of RK-13 cells was grown to confluence (defined as a continuous cell monolayer) and split into three separate flasks. These flasks were raised in parallel until confluence, when each was again split into two centrifuge tubes. Samples were spun at 400 g and trypsin removed. The cells were resuspended in 5 ml MEM. Spores were freshly harvested from 20 flasks of *T. hominis-*infected RK-13 cells and resuspended in 400 μl PBS (~2.3x10^7^ spores/ml). 100 μl of this spore suspension was added to one of the two centrifuge tubes containing RK-13 cells. Cells were incubated for 10 min before seeding to a new 175 cm^2^ flask containing 40 ml MEM.

Flasks of uninfected and *T. hominis* infected RK-13 cells were raised in parallel for 7 days post inoculation. During this time they were trypsinised and split twice in order to boost levels of infection. Two days after the final trypsinisation the cells were harvested in RNAprotect cell reagent and immediately frozen at −20 °C. At this stage, approximately 60 % of cells in flasks to which spores had been added exhibited signs of infection.

### Preparation of RNA for sequencing

Cells suspended in RNAprotect (Qiagen) were thawed and pelleted by centrifugation at 400 g. Total RNA was purified from each sample using the standard TRIzol (Invitrogen) extraction protocol, with the addition of a bead beating step (three times for 20 s at 5 m/s, with 10 mins incubation on ice between beating). An additional cleanup step using the GeneJet RNA purification kit (Thermo Scientific) was added to remove residual organic solvents from the purified total RNA. The RNA integrity and concentration was assessed using the Agilent RNA 6000 Nano Kit on the Agilent 2100 BioAnalyser. PolyA RNA was isolated from 5 μg of purified total RNA. Libraries were prepared using the ScriptSeq™ v2 RNA-Seq Library Preparation Kit (Epicentre Biotechnologies) and sequenced on the Illumina HiSeq 2500 in Rapid-Run mode, producing non-strand-specific 100 bp single-ended reads, with each library sequenced on two different lanes of the sequencer.

### Processing and analysis of RNA-seq data

The *Trachipleistophora hominis* genome and annotation [10] were obtained from NCBI, whilst the genome and annotation of the European rabbit (*Oryctolagus cuniculus:* GCA_000003625.1) were obtained from the Ensembl database. Bowtie2 [99] was used to separately index the genomes of *T. hominis* and *O. cuniculus.* Quality control on the raw RNA sequencing reads was performed using FastQC [100] and Illumina sequencing adapters and low quality bases were trimmed using fastq-mcf [101]. In order to quantify the expression levels of *T. hominis* transcripts, and for novel transcript discovery, TopHat2 [102] was used to map quality-filtered reads from each infected sample to the *T. hominis* genome. Transcripts were assembled using cufflinks [103]. The final transcriptome assembly was generated using cuffmerge [103]. All sequence data associated with this project has been deposited at NCBI under the BioProject ID PRJNA 278775. Linear mixed-effect models were used to assess differential expression between different categories of genes within the parasite transcriptome. We fit functional category as a fixed effect, with random effects for gene, technical replicate, and biological replicate, and used log FPKM values as the response variable.

Transcripts that mapped to unannotated regions of the *T. hominis* genome were screened for potential ORFs by using BLASTx to search against the nr database with an E-value threshold of 0.01. SNPs were identified using SamTools mpileup and bcftools [104]. Vcfutils.pl varFilter was applied under default settings to remove low quality SNPs, with the addition of a minimum read depth of 10 [105]. An additional filter was applied to remove bases with low mapping quality scores. Values of 20, 40, 60 and 80 were tested. In all cases application of the filter reduced non-peak (0.5 frequency) signals while retaining the overall distribution of the allele frequency spectrum. We used a 60 as a balance between stringently filtering out low quality mapping and retaining data. The location of SNPs relative to annotated *T. hominis* genes and the impact of SNPs on protein-coding sequences were assessed using SNPeff [106] and processed using SNPsift [107].

To maximise the data available for intron detection, reads from all samples including *T. hominis-*infected cells were pooled and mapped onto the *T. hominis* reference genome using TopHat2 [102]. The intron junctions identified from this mapping were manually filtered so as to retain only those junctions covered by more than one read. Overlapping junctions were merged.

For quantification of *O. cuniculus* transcripts, TopHat2 [102] was used to map reads from all samples from one lane of the sequencer to the *O. cuniculus* genome, and transcripts were assembled and quantified using cufflinks. The abundance of transcripts in the three flasks of uninfected RK-13 cells was compared to that in the three flasks of *T. hominis* infected RK-13 cells using cuffdiff [108]. All RNA sequencing results were analysed in R using the cummeRbund package [109]. KEGG pathway [87] assignment for significantly differentially expressed genes and pathway enrichment analysis was carried out using KOBAS 2.0 [88].

### Phylogenetic analysis of PiggyBac elements

We BLASTed *T. hominis* PiggyBac elements THOM_1159, THOM_1429 and the additional family member newly identified in our transcriptome against the nr database, retrieving the top 100 significant hits with an E-value of less than 0.05. Duplicate hits were manually removed before sequences were aligned using M-Coffee [110], combining the results of alignments using Muscle [111], Mafft [112], ProbCons [113], PCMA [114], and Fsa [115]. Poorly-aligning regions were identified and removed using trimAl [116]. Our phylogeny was inferred using the C20 model [117] implemented in PhyloBayes 3.3 [118].

### Assessing heterogeneous expression in duplicated gene families

The expanded gene families in *T. hominis* and other Microsporidia identified by Nakjang et al. [11] where *T. hominis* had at least two duplicate copies were investigated to compare the expression of individual genes. To identify the *T. hominis* gene families showing the greatest level of between-paralogue expression level divergence, we normalised the expression level of each gene by the average level of expression for the family to which it belonged, then took the standard deviation of these values for each family. We then ranked families by this score to identify the families with the greatest within-family divergence.

### Microscopy of *T. hominis* infected RK-13 cells

For phase contrast microscopy cells were fixed in a 50 % methonal 50 % acetone solution. After washing in water slides were mounted in Mowiol containing p-Phenylenediamine (0.01 %) which was allowed to set overnight at room temperature. and mounted in the same way. Microscopy was performed using the Zeiss Axio Imager II (Upright) in structured illumination (apotome) mode at the Newcastle University Bio-Imaging unit.

### Ethical approval

*T. hominis* was maintained in the RK-13 cell line so no ethical approval was required for this study.

## Availability of supporting data

All sequence data associated with this project has been deposited at NCBI under the BioProject ID PRJNA 278775, and the *Trachipleistophora hominis* genome assembly (GCA_000316135.1) has been updated with the new gene models identified here.

## Additional files

Additional file 1: Figure S1. Robustness and reproducibility of transcriptomic analysis for RK-13 cells during intracellular infection. Pairwise comparison of log_10_ FPKM values in rabbit kidney cell transcript quantification, as outlined in Fig. 2a for parasite transcripts. In this case biological replicates of uninfected RK-13 cells (samples labelled RK) are compared to those for T. hominis infected RK-13 cells (samples labelled TH). (TIFF 6369 kb) Additional file 2: Table S1. Expression levels of *T. hominis* transcripts. These include novel transcripts identified in this study (Locus tag labelled as “NA”). Genes annotated in the *T. hominis* genome but not detected during this study are also included for reference. In some cases multiple locus tags map to a single transcript, as discussed in the main text. (XLSX 486 kb) Additional file 3: Table S2. Predicted *T. hominis* intron junctions. The genomic location and sequencing depth of all intron junctions identified in the *T. hominis* genome by TopHat (see Methods). (XLSX 11 kb) Additional file 4: Figure S2. Alignment of a selection of identified intron sequences. An alignment of novel *T. hominis* intron sequences predicted from our transcriptome, suggesting no conserved intron motif. The alignment was generated using MUSCLE [111] and visualised using SeaView [120]. (PDF 62 kb) Additional file 5: Figure S3. Overall expression profile of the *T. hominis* transcriptome. Distribution of ranked FPKM values for the *T. hominis* transcriptome. The 95th percentile (2240 FPKM) is marked by a blue line and the mean expression value (705 FPKM) is marked by a red line. (PDF 30 kb) Additional file 6: Table S3. Heterogeneity in expression of duplicated gene families. All duplicated gene families [11] containing at least two paralogues in *T. hominis* ranked according to their expression heterogeneity index (see Methods). (XLSX 25 kb) Additional file 7: Figure S4. Heterogeneous expression in expanded microsporidian gene families. Distribution of ranked heterogeneity index (see Methods). The red line denotes the inflection point above which we considered gene families to show high levels of expression heterogeneity. (PDF 18 kb) Additional file 8: Figure S5. Phylogenetic analysis of PiggyBac transposons suggests a natural insect host for *T. hominis*– full tree. An expanded version of the tree from Figure 4 including GI accessions for all included sequences. The accession number of the newly identified *T. hominis* sequence (Clade B) is XLOC_002128. (PDF 22 kb) Additional file 9: Table S4. Expression levels of RK-13 transcripts. Where possible these were annotated with Ensembl gene names. For unannotated transcripts identified in this study Ensembl Gene IDs are labelled as “NA”. Genes annotated in the *O. cuniculus* genome with no detectable expression during this study are also included for reference. Due to overlapping genes and alternative splicing, some transcriptome gene IDs are associated with multiple Ensembl gene IDs and/or multiple Ensembl Protein IDs. (XLSX 8052 kb) Additional file 10: Table S5. RK-13 transcripts identified as significantly differentially expressed during infection. Genes identified by cuffdiff [108] as significantly differentially expressed in the RK-13 host cell during *T. hominis* infection. Q-values are P-values that have been corrected for multiple testing using the false discovery rate method [108]. (XLSX 345 kb) Additional file 11: Table S6. Testing for enrichment of differentially expressed genes in KEGG pathways. Fischer’s exact test with Benjamini and Hochberg correction [121] for the enrichment of genes identified as significantly differentially expressed in KEGG pathways in the RK-13 cell line. (XLSX 70 kb) Additional file 12: Figure S6. Host cell gene expression changes in KEGG pathways enriched for differentially expressed genes. Genes highlighted in cyan are significantly up-regulated upon infection with *T. hominis*, genes in red are down-regulated, and the expression of genes in purple does not significantly change. Genes were assigned to KEGG pathways [87] using the KOBAS annotation pipeline [88], and colours representing differential expression were assigned using the KEGG web server [122]. (PDF 613 kb)

## Abbreviations

FPKM: Fragments per kilobase of transcript per million mapped reads
NTT: Nucleotide transport protein
ORF: Open reading frame
RK: Rabbit kidney
SNP: Single nucleotide polymorphism
UTR: Untranslated region
