# Supplementary figures and images for: Transcriptomic profiling of host-parasite interactions in the microsporidian Trachipleistophora hominis
Source: BMC Genomics. 2015 Nov 21;16:983. doi: 10.1186/s12864-015-1989-z (PMC4654818; doi:10.1186/s12864-015-1989-z)

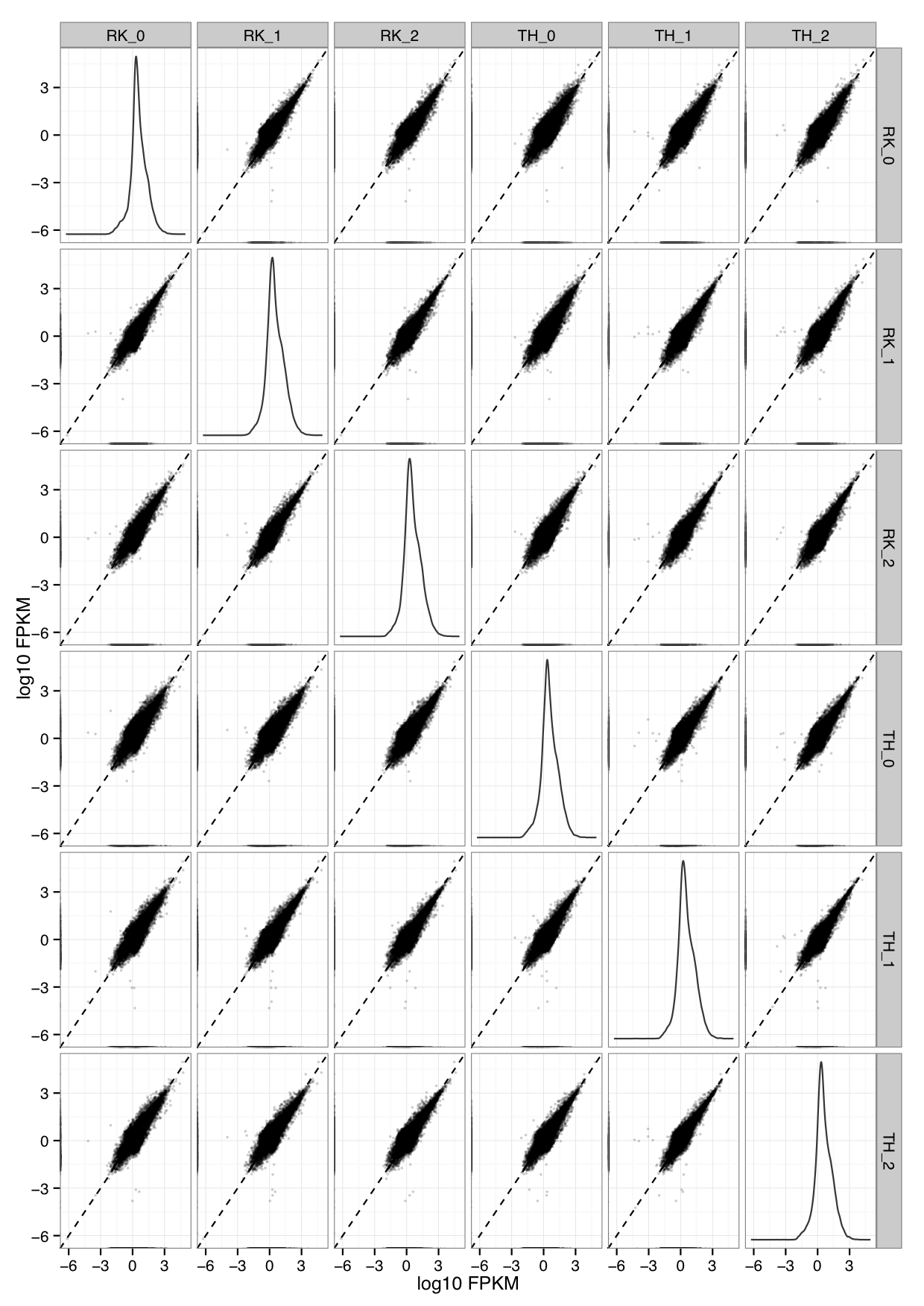

Supplement: Additional file 1: Figure S1. — Robustness and reproducibility of transcriptomic analysis for RK-13 cells during intracellular infection. Pairwise comparison of log10 FPKM values in rabbit kidney cell transcript quantification, as outlined in Fig. 2a for parasite transcripts. In this case biological replicates of uninfected RK-13 cells (samples labelled RK) are compared to those for T. hominis infected RK-13 cells (samples labelled TH). (TIFF 6369 kb) [file 12864_2015_1989_MOESM1_ESM.tiff]

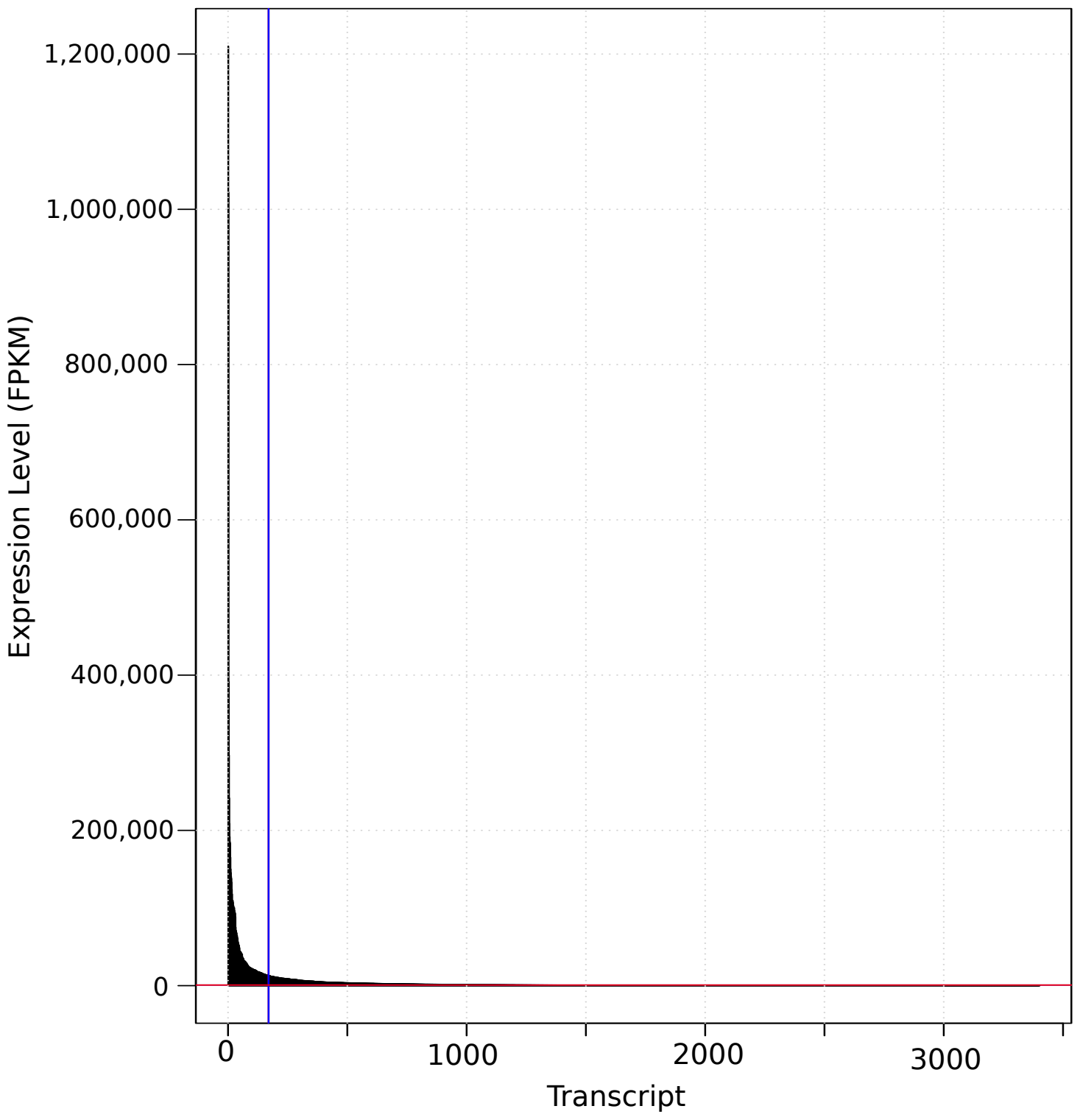

Supplement: Additional file 5: Figure S3. — Overall expression profile of the T. hominis transcriptome. Distribution of ranked FPKM values for the T. hominis transcriptome. The 95th percentile (2240 FPKM) is marked by a blue line and the mean expression value (705 FPKM) is marked by a red line. (PDF 30 kb) [file 12864_2015_1989_MOESM5_ESM.pdf]

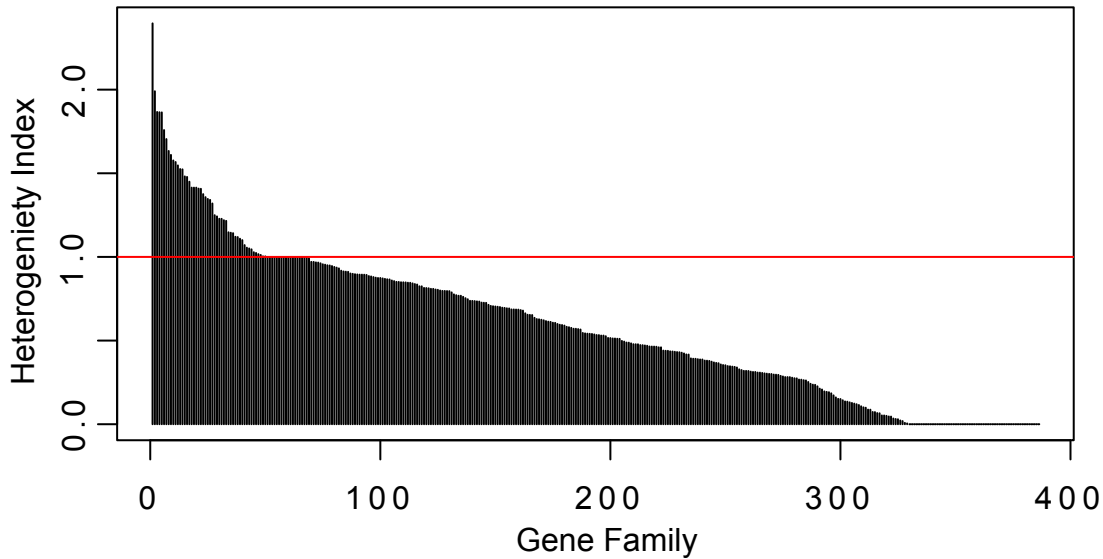

Supplement: Additional file 7: Figure S4. — Heterogeneous expression in expanded microsporidian gene families. Distribution of ranked heterogeneity index (see Methods). The red line denotes the inflection point above which we considered gene families to show high levels of expression heterogeneity. (PDF 18 kb) [file 12864_2015_1989_MOESM7_ESM.pdf]
