# Supplementary material for: Transcriptomic profiling of host-parasite interactions in the microsporidian Trachipleistophora hominis
Source: BMC Genomics. 2015 Nov 21;16:983. doi: 10.1186/s12864-015-1989-z (PMC4654818; doi:10.1186/s12864-015-1989-z)
Supplement: Additional file 4: Figure S2. — Alignment of a selection of identified intron sequences. An alignment of novel T. hominis intron sequences predicted from our transcriptome, suggesting no conserved intron motif. The alignment was generated using MUSCLE [111] and visualised using SeaView [120]. (PDF 62 kb) [file 12864_2015_1989_MOESM4_ESM.pdf]

Alignment: /Users/Andrew/junctions_files_TH_only/Sup_Figure_Junctions.fasta_aln
Seaview [blocks=10 fontsize=10 A4] on Mon Jun 30 03:55:06 2014

```
             1
JUNC00000002 ---------- ---------- ---------- ---------- ------ACGC CATAAAGTAC TACCGTCGCA
JUNC00000006 ---------- ---------- ---------- TTGAAAAACA AAAAGTAGTC CATCACGGCA TACCAGCGGT
JUNC00000009 ---------- ---------- ---------- ---------- ---------- ---CTAACCT TAATAGCCCT
JUNC00000011 GCTGTTGTCA ATGATGCTCT CATTCCATCC CTTTAAATCC TCACACAGCT CCTCTTTCTC CATCTTC---
JUNC00000013 ---------- ---------- ---------- ---------- ---------- ---------- ----GAAGAT
JUNC00000044 ---------- ---------- ---------- ---------- -------TGT TTGGAAAGTC GAGCGGTTCT
JUNC00000048 ---------- ---------- --TTCCATCG CTTTCTCAGC AAAATCAGGA CTCGTAAATC CTACAAAACC
JUNC00000054 ---------- ---------- ---------- ---------- ---------- ---------- ----------
JUNC00000056 ---------- ---------- ---------- ---------- ---------- ---------- CGCAGGAAGA
JUNC00000058 ---------- ---------- ---------- ---------- ---------- ---------T TATCA-----
JUNC00000060 ---------- ---------- ---------- TGTGGACAGA GCAAGAAGGA ---------- GGATGAAGAG
JUNC00000062 ---------- ---------- ---------- ---------- ---------- ------AATT TGTTGGTCTG

            71
JUNC00000002 AGAAGAGGGG AA-------- ---CCGCTTA TCACGA---- --AGCTCCGA TAGGTAAGGC CCCCTTCGCA
JUNC00000006 GGAGAAGGTT GTTATGCCTA ---CGGTTGA AGACAAAGAA TTGGTACTGA AAAATCAAGA ACCTAAAAAA
JUNC00000009 AGTAAATCTA ACCTAACCTC TAACCCTAAG TCAAACCCTA ATTCTACTAA CCCTCTAAAC CCTA------
JUNC00000011 ---------- -TCGCCCATC ---CCGTCTT TCATATGCTG CCCGTCCTG- TGCATGATAC TCCCTGCGCT
JUNC00000013 TAAGGGGGTT GAACGCCTTA GAGACACCGT TAATTATTTT TACGGGTTGA TTGGTAAAGT AGAGAGCGTT
JUNC00000044 GGTGAAGAAG ATG------- ---ACGAGAA AAAGGGTGGA TCAGGATTAA CAGGTA---- ----------
JUNC00000048 CAAGAAAACG GTCTCACCGT ---TCCTCAA TCGCTT---G TCAGTCTTCA AAAG------ ----------
JUNC00000054 ---------- ---------- ---------- ---------- -------TCC TGAATTGATA CCCTCTCTTT
JUNC00000056 AGAGGAGGAG GGGCTGTCCA ---CAAATTA CGAAAA---- -------TGA AAAAACGA-- ----------
JUNC00000058 ---------- --TTTCCTTC TACCTTATCA TGGCCTTGTC CTTCTACCTG ATTGTCAGTA CCTT------
JUNC00000060 AAGAAGAAAG GCAAGCCTTG ---TCCTGAG AAAGGAGGAG TATGCAGTGA GAAGGGCAAG CCCT------
JUNC00000062 GTTGGGCGGG ATTCAACTTT ---TT--TGA CGGGGCTTGG CTGGTGGTGA A--------- ----------

           141
JUNC00000002 CGTGGCATAG TGCTAGAT-A AAACGTAAGT GATTCTTAAT TTAGATAGGG ACA------- ----------
JUNC00000006 CTTG---TAA TTAAAGAA-A AACCGGTCGC TAA------- --GACGAAGG AAA------- ----------
JUNC00000009 ---------- ---------A TTCTACTAAC CCCTCTAACC CTAATCA--- ---------- ----------
JUNC00000011 TGTACCCCTC GTCATCAA-A GCCTCTGCGC TCACGTGTGT AGTACGGCCT GCACCCGATC TGCATGTTCT
JUNC00000013 GATGTAGGGG GTAACGAGGA TTATAAGGGT GTGGTTTATA AGGAAGGGGT A--------- ----------
JUNC00000044 ---------- ---------A GTCTGGCGGT TCTGGTGAGG ATGATGACGA GAA------- ----------
JUNC00000048 -GTTACTTAA GTTAGTATCA GAACTTTCGT TCATCTCCGC TCAGTTACAC GAA------- ----------
JUNC00000054 CTTCGCCTGT GTTAACGCCT TCTCTTCCCT CGTCCTC--- ---------- ---------- ----------
JUNC00000056 -CTGTAATAG AAGAAAAG-A TACCGGCGGG CGAGCTGGTC ATAGAGGAGG AAA------- ----------
JUNC00000058 ------CTAC CTTATCATGG CCTTGTCCTC CTACCTGATT ATCAGTACCT TCT------- ----------
JUNC00000060 ----GCTCAG ATAAAGACGA GCCTTGTCCC TGTCCTGAGA AAGGAGGAGT ATG------- ----------
JUNC00000062 -------TGA CTTATTAC-G TTTTGCGGTC TGATCCTTCT CTGGCAATGT GCT------- ----------

           211
JUNC00000002 ------TCTG AAAATGATGA ATCTATGGTA TGATACTAAC TTTC------ ---------- ----------
JUNC00000006 ---------- ---------- --TAGTGGTA GAGCAAGAGC CGTC------ ---------- ----------
JUNC00000009 ------TCCT ACTAACCCTA ATCCAAGGTC TAACCCCATT CCTAAT---- ---------- ----------
JUNC00000011 TAAACGTAAC GTTGTCAAGT ATCCTTAACG CGTCCTCCTT CGCCTCTATC GAGCCGAACT CTACGATCCC
JUNC00000013 ---------- ---------- GTTAATAAGG ATGTGGAGGG TAAG------ ---------- ----------
JUNC00000044 ---------- ---------- --AAAGGATG GATCAGGATT AACA------ ---------- ----------
JUNC00000048 ---------- -----CCAAG GTTGAAATTA AATCATCACT TACATTTCGC ---------- ----------
JUNC00000054 ---------- ---------- ATTGATACCC TCTCTTCCTT CGCC------ ---------- ----------
JUNC00000056 ------TGGT GCACAAAGAA ATTGTTAGAG AGACAGTAAG CATC------ ---------- ----------
JUNC00000058 ---------- -----ACCTT ATCAACCTCA TTACATTTTT CAAC------ ---------- ----------
JUNC00000060 CAGTGAGAAA GACAAACCAT GCTCAGATAA AGACAAGCCT TGTC------ ---------- ----------
JUNC00000062 ---------G GTGGTGAATG ATTTATTACG TTTTACGGTC TGAT------ ---------- ----------

           281
JUNC00000002 ---------- ---------- ---------- ---------- --------AG TGGTGTAGAA GCGAAACAGC
JUNC00000006 ---------- ---------- ---------- ---------- ---------- -GTCAATGAA AAG-------
JUNC00000009 ---------- ---------- ---------- ---------- --------CC TACTATCACT ACA-------
JUNC00000011 GTGCTGACCA GACTGCGTAA TACGCGCATA CACGGGGTCT AACCGGAACG TACGCACGAA ATCCTTGAGC
JUNC00000013 ---------- ---------- ---------- ---------- --------GG TAAGAATGAG GGGATGGAAG
JUNC00000044 ---------- ---------- ---------- ---------- ---------- ---------- ----------
JUNC00000048 ---------- ---------- ---------- ---------- --------CA TATTTGCTGA ATG-------
JUNC00000054 ---------- ---------- ---------- ---------- --------CG TGTTAACGCC TTCTCTTCCC
JUNC00000056 ---------- ---------- ---------- ---------- --------GA TACATTCACA AAA------G
JUNC00000058 ---------- ---------- ---------- ---------- --------CT TATCTACGCA TCCTATCAAA
JUNC00000060 ---------- ---------- ---------- ---------- --------CC TGTCCTGAGA A---------
JUNC00000062 ---------- ---------- ---------- ---------- --------CC TTCTCTGGCA ATG-------
```

```
              351
JUNC00000002  CCAACTCAGC TGTCCGTAAG GCTGTTAGGA TACAGCTCTG CGGAACAGGA AAGAAAAT-- -AATTGCATT
JUNC00000006  -GGTAACTGT AGATAGCGAA CCCGATGTCA CACTT----- -------GTA AAGCCTGCTC AAACCACAGA
JUNC00000009  ---------- ---------- ---------- ---------- ---------- ---------- ----------
JUNC00000011  TCAATCACGT TGCACTCGGG GATGTTGTCG AGTACCACCT TGAACTTGTC AAGGGGGTGC CCGTTGCGCT
JUNC00000013  GGGTAATTAA TAAGGGTGTG GTTTATAAGG AAGGGGTAAT TAATAATGAG AGGGTGATTA ATAATGAGGA
JUNC00000044  ---------- GGTAAGTCTG GCGGTTCTGG TGAAGAAGAT GATGAGAAAA AGGATGGATC AGGATTGA--
JUNC00000048  ---------- ---TGGCAAG GAGGTCGCCA ACCTTTGCCT CAGGAGAGAG ATCGCTGATA ACAATGGTCC
JUNC00000054  TCGCCCTCAT TGATACC--- ---------- ---------- ---------- ---------- ----------
JUNC00000056  GTAGGATAGC AGAGGATATA GCCGGTAATT TGAAG----- -------GAA GAGATGCGAA ATGATCTAAA
JUNC00000058  GGACTAATCC TTACCATCTC CCCATTATAA TAATA----- ---------- ---------- ----------
JUNC00000060  ---------- ---------- ---------- ---------- ---------- ---------- ----------
JUNC00000062  -------TGC TGGTGTTTTG GTTGCTACTT ---------- ----ACAGAT GAATTTGTTC GTTTTATATT

              421
JUNC00000002  CGTGCCGTAC GA-------- ---------- ---------- ---------- -
JUNC00000006  GGTGGTCAAT GTCCCGGG-- ---------- ---------- ---------- -
JUNC00000009  ---------- ---------- ---------- ---------- ---------- -
JUNC00000011  GCACCCTCTC GTGGTATCTA TCGTCAGCCT TGTTGTGCAT GAACTCGCGT T
JUNC00000013  GGTAGTCTAT AATGAAGG-- ---------- ---------- ---------- -
JUNC00000044  ---------- ---------- ---------- ---------- ---------- -
JUNC00000048  TTTTTTTCTT GTTTTC---- ---------- ---------- ---------- -
JUNC00000054  ---------- ---------- ---------- ---------- ---------- -
JUNC00000056  AGTAGGATTT G--------- ---------- ---------- ---------- -
JUNC00000058  ---------- ---------- ---------- ---------- ---------- -
JUNC00000060  ---------- ---------- ---------- ---------- ---------- -
JUNC00000062  TTTATCTTTC TTGACGGGCA CATTCACAGT TTTAGGCTCC T--------- -
```
