# Supplementary material for: Transcriptomic profiling of host-parasite interactions in the microsporidian Trachipleistophora hominis
Source: BMC Genomics. 2015 Nov 21;16:983. doi: 10.1186/s12864-015-1989-z (PMC4654818; doi:10.1186/s12864-015-1989-z)
Supplement: Additional file 8: Figure S5. — Phylogenetic analysis of PiggyBac transposons suggests a natural insect host for T. hominis– full tree. An expanded version of the tree from Figure 4 including GI accessions for all included sequences. The accession number of the newly identified T. hominis sequence (Clade B) is XLOC_002128. (PDF 22 kb) [file 12864_2015_1989_MOESM8_ESM.pdf]

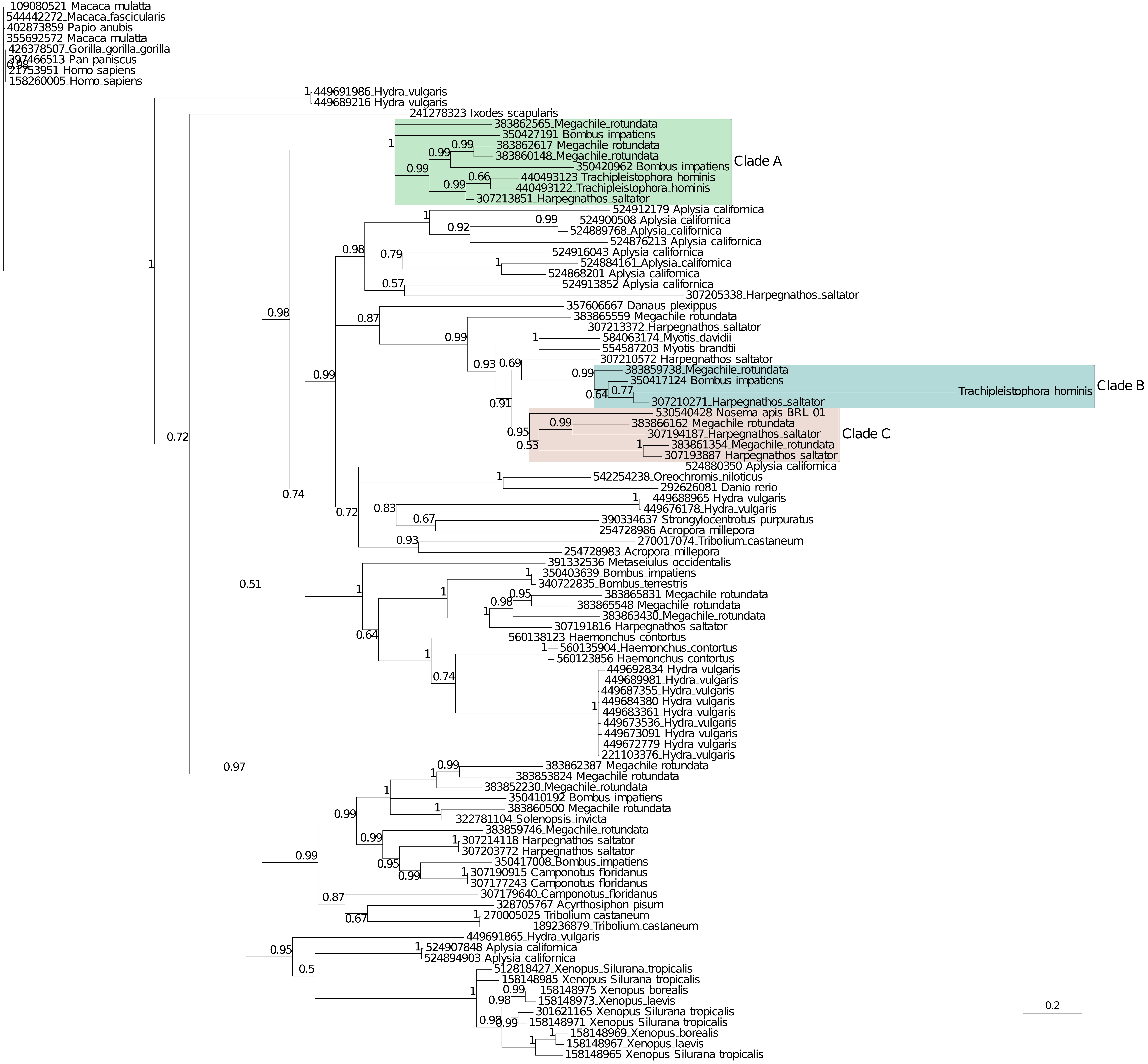
109080521_Macaca_mulatta
544442272_Macaca_fascicularis
402873859_Papio_anubis
355692572_Macaca_mulatta
426378507_Gorilla_gorilla_gorilla
397466513_Pan_paniscus
21753951_Homo_sapiens
158260005_Homo_sapiens
449691986_Hydra_vulgaris
449689216_Hydra_vulgaris
241278323_Ixodes_scapularis
383862565_Megachile_rotundata
350427191_Bombus_impatiens
383862617_Megachile_rotundata
383860148_Megachile_rotundata
350420962_Bombus_impatiens
440493123_Trachipleistophora_hominis
440493122_Trachipleistophora_hominis
307213851_Harpegnathos_saltator
Clade A
524912179_Aplysia_californica
524900508_Aplysia_californica
524889768_Aplysia_californica
524876213_Aplysia_californica
524916043_Aplysia_californica
524884161_Aplysia_californica
524868201_Aplysia_californica
524913852_Aplysia_californica
307205338_Harpegnathos_saltator
357606667_Danaus_plexippus
383865559_Megachile_rotundata
307213372_Harpegnathos_saltator
584063174_Myotis_davidii
554587203_Myotis_brandtii
307210572_Harpegnathos_saltator
383859738_Megachile_rotundata
350417124_Bombus_impatiens
Trachipleistophora_hominis
307210271_Harpegnathos_saltator
Clade B
530540428_Nosema_apis_BRL_01
383866162_Megachile_rotundata
307194187_Harpegnathos_saltator
383861354_Megachile_rotundata
307193887_Harpegnathos_saltator
Clade C
524880350_Aplysia_californica
542254238_Oreochromis_niloticus
292626081_Danio_rerio
449688965_Hydra_vulgaris
449676178_Hydra_vulgaris
390334637_Strongylocentrotus_purpuratus
254728986_Acropora_millepora
270017074_Tribolium_castaneum
254728983_Acropora_millepora
391332536_Metaseiulus_occidentalis
350403639_Bombus_impatiens
340722835_Bombus_terrestris
383865831_Megachile_rotundata
383865548_Megachile_rotundata
383863430_Megachile_rotundata
307191816_Harpegnathos_saltator
560138123_Haemonchus_contortus
560135904_Haemonchus_contortus
560123856_Haemonchus_contortus
449692834_Hydra_vulgaris
449689981_Hydra_vulgaris
449687355_Hydra_vulgaris
449684380_Hydra_vulgaris
449683361_Hydra_vulgaris
449673536_Hydra_vulgaris
449673091_Hydra_vulgaris
449672779_Hydra_vulgaris
221103376_Hydra_vulgaris
383862387_Megachile_rotundata
383853824_Megachile_rotundata
383852230_Megachile_rotundata
350410192_Bombus_impatiens
383860500_Megachile_rotundata
322781104_Solenopsis_invicta
383859746_Megachile_rotundata
307214118_Harpegnathos_saltator
307203772_Harpegnathos_saltator
350417008_Bombus_impatiens
307190915_Camponotus_floridanus
307177243_Camponotus_floridanus
307179640_Camponotus_floridanus
328705767_Acyrthosiphon_pisum
270005025_Tribolium_castaneum
189236879_Tribolium_castaneum
449691865_Hydra_vulgaris
524907848_Aplysia_californica
524894903_Aplysia_californica
512818427_Xenopus_Silurana_tropicalis
158148985_Xenopus_Silurana_tropicalis
158148975_Xenopus_borealis
158148973_Xenopus_laevis
301621165_Xenopus_Silurana_tropicalis
158148971_Xenopus_Silurana_tropicalis
158148969_Xenopus_borealis
158148967_Xenopus_laevis
158148965_Xenopus_Silurana_tropicalis
0.2
