# Supplementary material for: Transcriptomic profiling of host-parasite interactions in the microsporidian Trachipleistophora hominis
Source: BMC Genomics. 2015 Nov 21;16:983. doi: 10.1186/s12864-015-1989-z (PMC4654818; doi:10.1186/s12864-015-1989-z)
Supplement: Additional file 12: Figure S6. — Host cell gene expression changes in KEGG pathways enriched for differentially expressed genes. Genes highlighted in cyan are significantly up-regulated upon infection with T. hominis, genes in red are down-regulated, and the expression of genes in purple does not significantly change. Genes were assigned to KEGG pathways [87] using the KOBAS annotation pipeline [88], and colours representing differential expression were assigned using the KEGG web server [122]. (PDF 613 kb) [file 12864_2015_1989_MOESM12_ESM.pdf]

# OXIDATIVE PHOSPHORYLATION

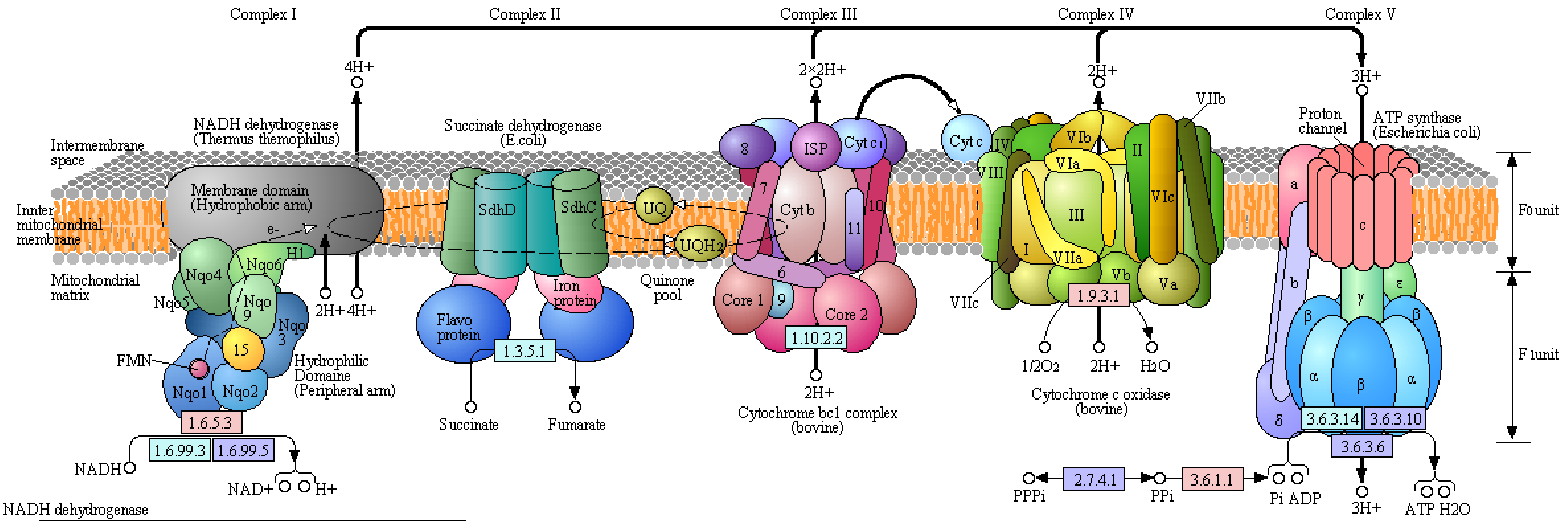


NADH dehydrogenase

| | | | | | | | | | | | | | | | | | |
|---|---|---|---|---|---|---|---|---|---|---|---|---|---|---|---|---|---|
| E | ND1 | ND2 | ND3 | ND4 | ND4L | ND5 | ND6 | | | | | | | | | | |
| E | Ndufs1 | Ndufs2 | Ndufs3 | Ndufs4 | Ndufs5 | Ndufs6 | Ndufs7 | Ndufs8 | Ndufv1 | Ndufv2 | Ndufv3 | | | | | | |
| B/A | NuoA | NuoB | NuoC | NuoD | NuoE | NuoF | NuoG | NuoH | NuoI | NuoJ | NuoK | NuoL | NuoM | NuoN | | | |
| B/A | NdhC | NdhK | NdhJ | NdhH | NdhA | NdhI | NdhG | NdhE | NdhF | NdhD | NdhB | NdhL | NdhM | NdhN | HoxE | HoxF | HoxU |
| E | Ndufa1 | Ndufa2 | Ndufa3 | Ndufa4 | Ndufa5 | Ndufa6 | Ndufa7 | Ndufa8 | Ndufa9 | Ndufa10 | Ndufab1 | Ndufa11 | Ndufa12 | Ndufa13 | | | |
| E | Ndufb1 | Ndufb2 | Ndufb3 | Ndufb4 | Ndufb5 | Ndufb6 | Ndufb7 | Ndufb8 | Ndufb9 | Ndufb10 | Ndufb11 | Ndufc1 | Ndufc2 | | | | |

Succinate dehydrogenase / Fumarate reductase

| | | | | | |
|---|---|---|---|---|---|
| E | SDHC | SDHD | SDHA | SDHB | |
| B/A | SdhC | SdhD | SdhA | SdhB | |
| | | FrdA | FrdB | FrdC | FrdD |

Cytochrome c reductase

| | | | | | | | | | |
|---|---|---|---|---|---|---|---|---|---|
| E/B/A | ISP | Cytb | Cyt1 | | | | | | |
| E | | | | COR1 | QCR2 | QCR6 | QCR7 | QCR8 | QCR9 | QCR10 |

Cytochrome c oxidase

| | | | | | | | | | | | | | | | | | |
|---|---|---|---|---|---|---|---|---|---|---|---|---|---|---|---|---|---|
| E | COX10 | | COX3 | COX1 | COX2 | COX4 | COX5A | COX5B | COX6A | COX6B | COX6C | COX7A | COX7B | COX7C | COX8 | E/B/A: COX11 | COX15 | COX17 |
| B/A | CyoE | CyoD | CyoC | CyoB | CyoA | | | | | | | | | | | | | |
| | | CoxD | CoxC | CoxA | CoxB | | | | | | | | | | | | | |
| | | QoxD | QoxC | QoxB | QoxA | | | | | | | | | | | | | |

Cytochrome c oxidase , cbb3-type

| | | | | |
|---|---|---|---|---|
| B | I | II | IV | III |

Cytochrome bd complex

| | | |
|---|---|---|
| B/A | CydA | CydB |

F-type ATPase (Bacteria)

| | | | | |
|---|---|---|---|---|
| alpha | beta | gamma | delta | epsilon |
| a | b | c | | |

F-type ATPase (Eukaryotes)

| | | | | | |
|---|---|---|---|---|---|
| alpha | beta | gamma | delta | epsilon | |
| OSCP | a | b | c | d | e |
| f | g | f6/h | j | k | 8 |

V/A-type ATPase (Bacteria, Archaeas)

| | | | | | | |
|---|---|---|---|---|---|---|
| A | B | C | D | E | F | G/H |
| I | K | | | | | |

V-type ATPase (Eukaryotes)

| | | | | | | | |
|---|---|---|---|---|---|---|---|
| A | B | C | D | E | F | G | H |
| a | c | d | e | S1 | | | |

00190 5/7/14

(c) Kanehisa Laboratories

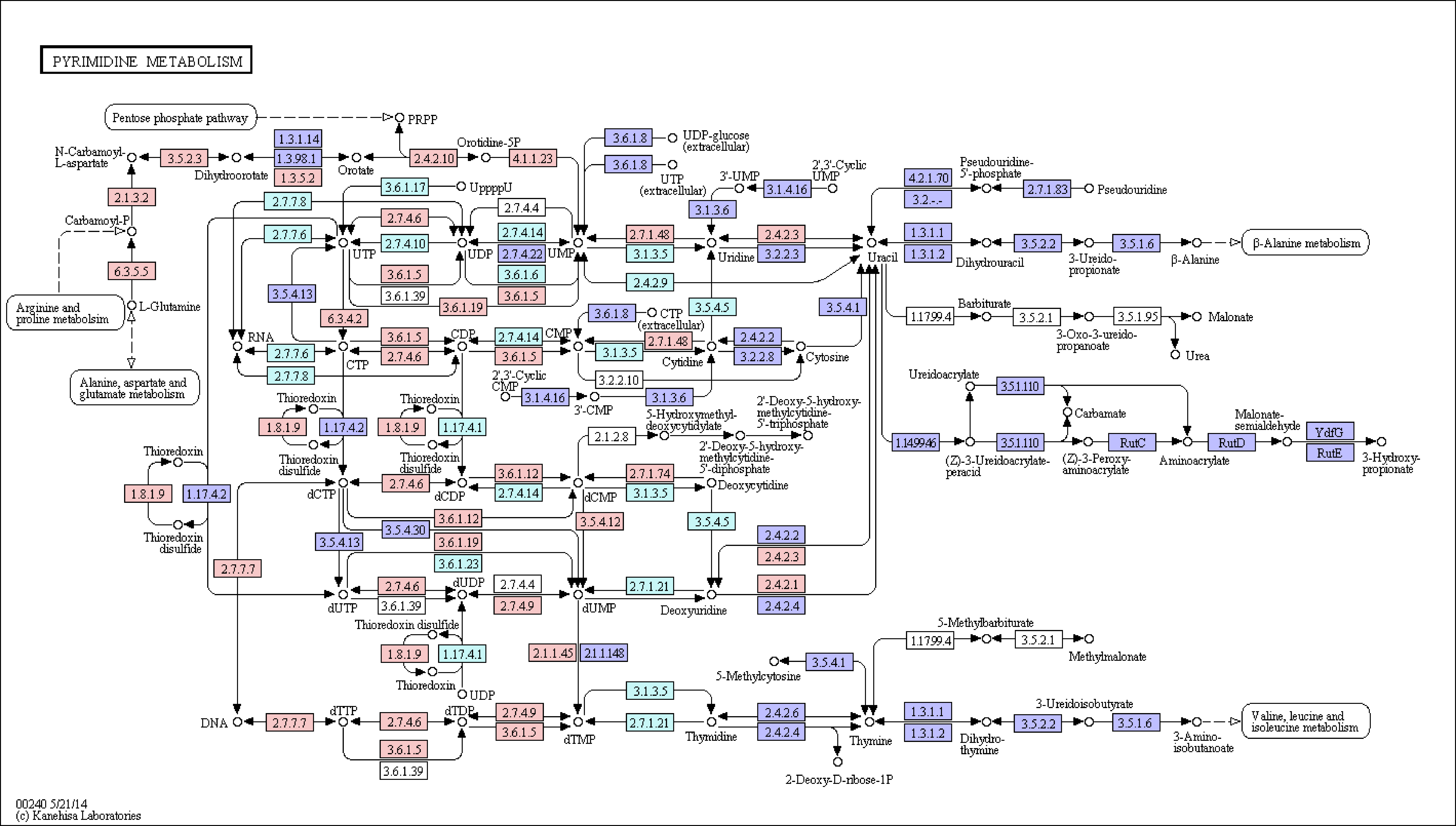
PYRIMIDINE METABOLISM
Pentose phosphate pathway
PRPP
N-Carbamoyl-L-aspartate
3.5.2.3
Dihydroorotate
1.3.1.14
1.3.98.1
1.3.5.2
Orotate
2.4.2.10
Orotidine-5P
4.1.1.23
2.1.3.2
Carbamoyl-P
6.3.5.5
L-Glutamine
Arginine and proline metabolsim
Alanine, aspartate and glutamate metabolism
3.6.1.17
UppppU
2.7.7.8
2.7.4.4
2.7.4.6
2.7.7.6
UTP
2.7.4.10
UDP
2.7.4.14
2.7.4.22
UMP
3.6.1.6
3.6.1.5
3.6.1.39
3.6.1.19
3.5.4.13
6.3.4.2
3.6.1.8
UDP-glucose (extracellular)
UTP (extracellular)
3'-UMP
3.1.4.16
2',3'-Cyclic UMP
3.1.3.6
2.7.1.48
3.1.3.5
Uridine
2.4.2.3
3.2.2.3
2.4.2.9
Uracil
3.5.4.5
3.5.4.1
4.2.1.70
3.2.-.-
Pseudouridine-5'-phosphate
2.7.1.83
Pseudouridine
1.3.1.1
1.3.1.2
Dihydrouracil
3.5.2.2
3-Ureido-propionate
3.5.1.6
β-Alanine
β-Alanine metabolism
1.17.99.4
Barbiturate
3.5.2.1
3-Oxo-3-ureido-propanoate
3.5.1.95
Malonate
Urea
RNA
CTP
CDP
CMP
CTP (extracellular)
2.4.2.2
3.2.2.8
Cytidine
Cytosine
3.2.2.10
2',3'-Cyclic CMP
3'-CMP
Thioredoxin
Thioredoxin disulfide
1.8.1.9
1.17.4.2
1.17.4.1
5-Hydroxymethyl-deoxycytidylate
2'-Deoxy-5-hydroxy-methylcytidine-5'-triphosphate
2'-Deoxy-5-hydroxy-methylcytidine-5'-diphosphate
2.1.2.8
Ureidoacrylate
3.5.1.110
Carbamate
1.14.99.46
(Z)-3-Ureidoacrylate-peracid
(Z)-3-Peroxy-aminoacrylate
RutC
Aminoacrylate
RutD
Malonate-semialdehyde
YdfG
RutE
3-Hydroxy-propionate
dCTP
dCDP
3.6.1.12
2.7.1.74
dCMP
Deoxycytidine
3.5.4.12
3.5.4.30
3.6.1.23
2.7.7.7
dUTP
dUDP
2.7.4.9
dUMP
2.7.1.21
Deoxyuridine
2.4.2.1
2.4.2.4
Thioredoxin disulfide
2.1.1.45
2.1.1.148
5-Methylbarbiturate
Methylmalonate
5-Methylcytosine
DNA
dTTP
dTDP
dTMP
Thymidine
2.4.2.6
Thymine
2-Deoxy-D-ribose-1P
Dihydro-thymine
3-Ureidoisobutyrate
3-Amino-isobutanoate
Valine, leucine and isoleucine metabolism
00240 5/21/14
(c) Kanehisa Laboratories

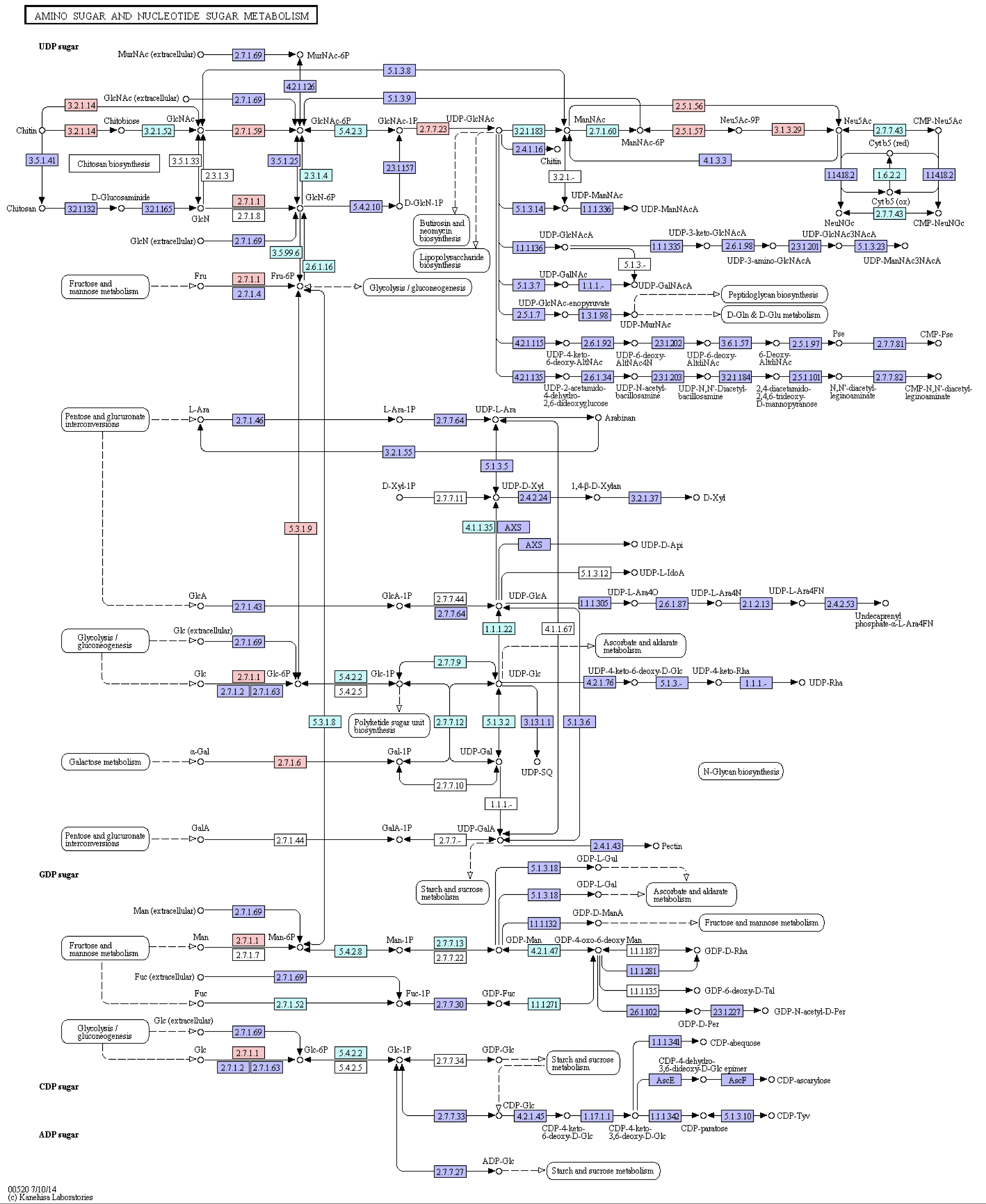

AMINO SUGAR AND NUCLEOTIDE SUGAR METABOLISM
UDP sugar
MurNAc (extracellular)
2.7.1.69
MurNAc-6P
5.1.3.8
4.2.1.126
GlcNAc (extracellular)
2.7.1.69
5.1.3.9
3.2.1.14
Chitobiose
GlcNAc
GlcNAc-6P
GlcNAc-1P
UDP-GlcNAc
ManNAc
Neu5Ac-9P
Neu5Ac
CMP-Neu5Ac
2.5.1.56
Chitin
3.2.1.14
3.2.1.52
2.7.1.59
5.4.2.3
2.7.7.23
3.2.1.183
2.7.1.60
2.5.1.57
3.1.3.29
2.7.7.43
ManNAc-6P
Cyt b5 (red)
2.4.1.16
Chitin
3.5.1.41
Chitosan biosynthesis
3.5.1.33
3.5.1.25
2.3.1.157
4.1.3.3
2.3.1.3
2.3.1.4
3.2.1.-
1.14.18.2
1.6.2.2
1.14.18.2
UDP-ManNAc
Cyt b5 (ox)
D-Glucosaminide
GlcN-6P
Chitosan
3.2.1.132
3.2.1.165
2.7.1.1
5.4.2.10
D-GlcN-1P
5.1.3.14
1.1.1.336
UDP-ManNAcA
2.7.7.43
GlcN
2.7.1.8
NeuNGc
CMP-NeuNGc
Butirosin and neomycin biosynthesis
GlcN (extracellular)
2.7.1.69
UDP-GlcNAcA
UDP-3-keto-GlcNAcA
UDP-GlcNAc3NAcA
1.1.1.136
1.1.1.335
2.6.1.98
2.3.1.201
5.1.3.23
3.5.99.6
Lipopolysaccharide biosynthesis
UDP-3-amino-GlcNAcA
UDP-ManNAc3NAcA
2.6.1.16
5.1.3.-
UDP-GalNAc
Fru
2.7.1.1
Fru-6P
Fructose and mannose metabolism
2.7.1.4
Glycolysis / gluconeogenesis
5.1.3.7
1.1.1.-
UDP-GalNAcA
Peptidoglycan biosynthesis
UDP-GlcNAc-enopyruvate
2.5.1.7
1.3.1.98
D-Gln & D-Glu metabolism
UDP-MurNAc
Pse
CMP-Pse
4.2.1.115
2.6.1.92
2.3.1.202
3.6.1.57
2.5.1.97
2.7.7.81
UDP-4-keto-6-deoxy-AltNAc
UDP-6-deoxy-AltNAc4N
UDP-6-deoxy-AltdiNAc
6-Deoxy-AltdiNAc
4.2.1.135
2.6.1.34
2.3.1.203
3.2.1.184
2.5.1.101
2.7.7.82
UDP-2-acetamido-4-dehydro-2,6-dideoxyglucose
UDP-N-acetyl-bacillosamine
UDP-N,N'-Diacetyl-bacillosamine
2,4-diacetamido-2,4,6-trideoxy-D-mannopyranose
N,N'-diacetyl-leginoaminate
CMP-N,N'-diacetyl-leginoaminate
Pentose and glucuronate interconversions
L-Ara
2.7.1.46
L-Ara-1P
2.7.7.64
UDP-L-Ara
Arabinan
3.2.1.55
5.1.3.5
D-Xyl-1P
UDP-D-Xyl
1,4-β-D-Xylan
2.7.7.11
2.4.2.24
3.2.1.37
D-Xyl
5.3.1.9
4.1.1.35
AXS
AXS
UDP-D-Api
5.1.3.12
UDP-L-IdoA
GlcA
GlcA-1P
2.7.7.44
UDP-GlcA
UDP-L-Ara4O
UDP-L-Ara4N
UDP-L-Ara4FN
2.7.1.43
2.7.7.64
1.1.1.305
2.6.1.87
2.1.2.13
2.4.2.53
Undecaprenyl phosphate-α-L-Ara4FN
Glycolysis / gluconeogenesis
Glc (extracellular)
2.7.1.69
1.1.1.22
4.1.1.67
Ascorbate and aldarate metabolism
2.7.7.9
Glc
2.7.1.1
Glc-6P
5.4.2.2
Glc-1P
UDP-Glc
UDP-4-keto-6-deoxy-D-Glc
UDP-4-keto-Rha
2.7.1.2
2.7.1.63
5.4.2.5
4.2.1.76
5.1.3.-
1.1.1.-
UDP-Rha
5.3.1.8
Polyketide sugar unit biosynthesis
2.7.7.12
5.1.3.2
3.13.1.1
5.1.3.6
Galactose metabolism
α-Gal
2.7.1.6
Gal-1P
UDP-Gal
UDP-SQ
N-Glycan biosynthesis
2.7.7.10
1.1.1.-
Pentose and glucuronate interconversions
GalA
2.7.1.44
GalA-1P
2.7.7.-
UDP-GalA
2.4.1.43
Pectin
GDP sugar
5.1.3.18
GDP-L-Gul
Starch and sucrose metabolism
GDP-L-Gal
5.1.3.18
Ascorbate and aldarate metabolism
Man (extracellular)
2.7.1.69
1.1.1.132
GDP-D-ManA
Fructose and mannose metabolism
Man
2.7.1.1
Man-6P
Man-1P
2.7.7.13
GDP-Man
GDP-4-oxo-6-deoxy Man
Fructose and mannose metabolism
2.7.1.7
5.4.2.8
2.7.7.22
4.2.1.47
1.1.1.187
GDP-D-Rha
1.1.1.281
Fuc (extracellular)
2.7.1.69
Fuc
Fuc-1P
GDP-Fuc
1.1.1.135
GDP-6-deoxy-D-Tal
2.7.1.52
2.7.7.30
1.1.1.271
2.6.1.102
2.3.1.227
GDP-N-acetyl-D-Per
GDP-D-Per
Glycolysis / gluconeogenesis
Glc (extracellular)
2.7.1.69
1.1.1.341
CDP-abequose
Glc
2.7.1.1
Glc-6P
5.4.2.2
Glc-1P
GDP-Glc
2.7.7.34
Starch and sucrose metabolism
CDP-4-dehydro-3,6-dideoxy-D-Glc epimer
2.7.1.2
2.7.1.63
5.4.2.5
AscE
AscF
CDP-ascarylose
CDP sugar
CDP-Glc
2.7.7.33
4.2.1.45
1.17.1.1
1.1.1.342
5.1.3.10
CDP-Tyv
CDP-4-keto-6-deoxy-D-Glc
CDP-4-keto-3,6-deoxy-D-Glc
CDP-paratose
ADP sugar
ADP-Glc
2.7.7.27
Starch and sucrose metabolism
00520 7/10/14
(c) Kanehisa Laboratories

# RIBOSOME

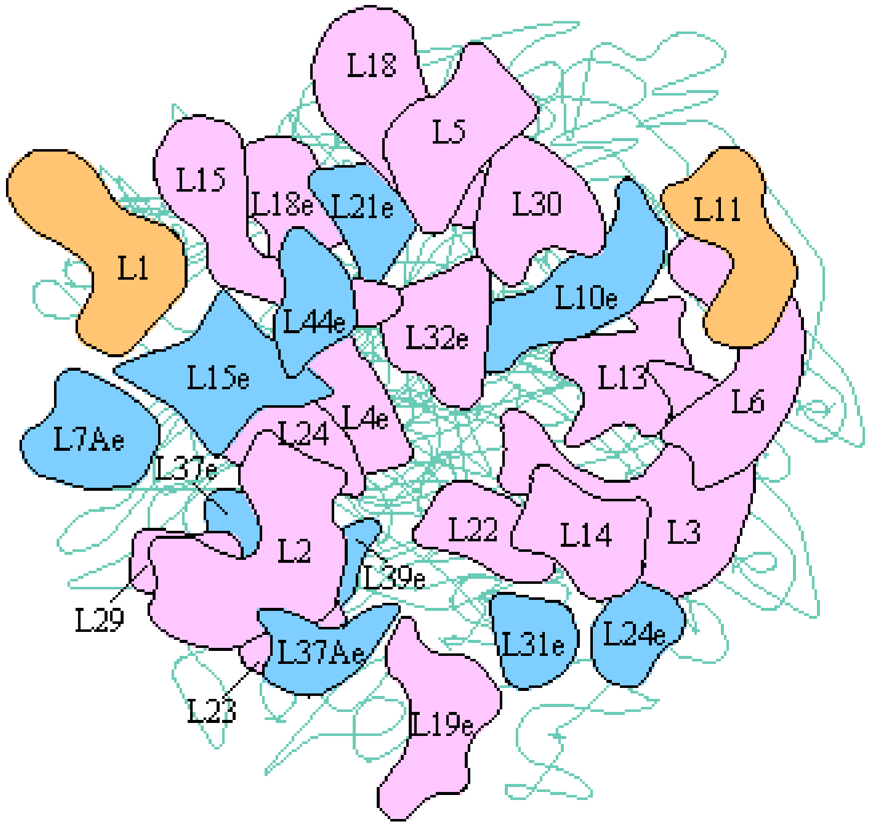


Large subunit (Haloarcula marismortui)

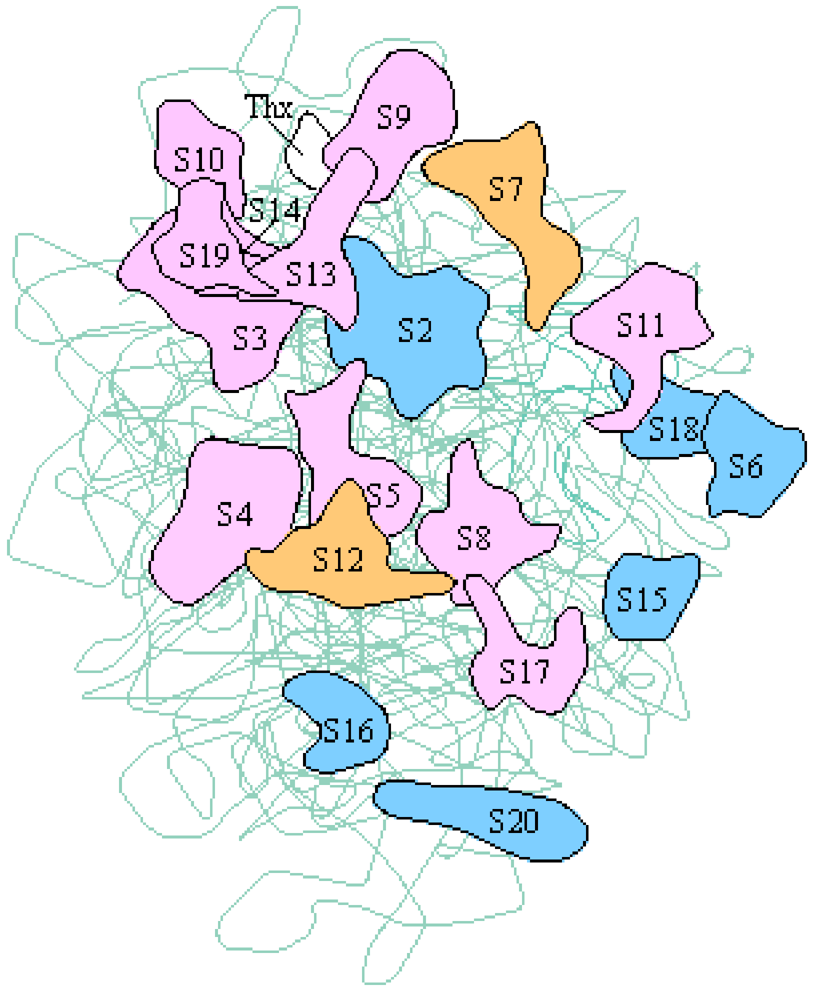


Small subunit (Thermus aquaticus)

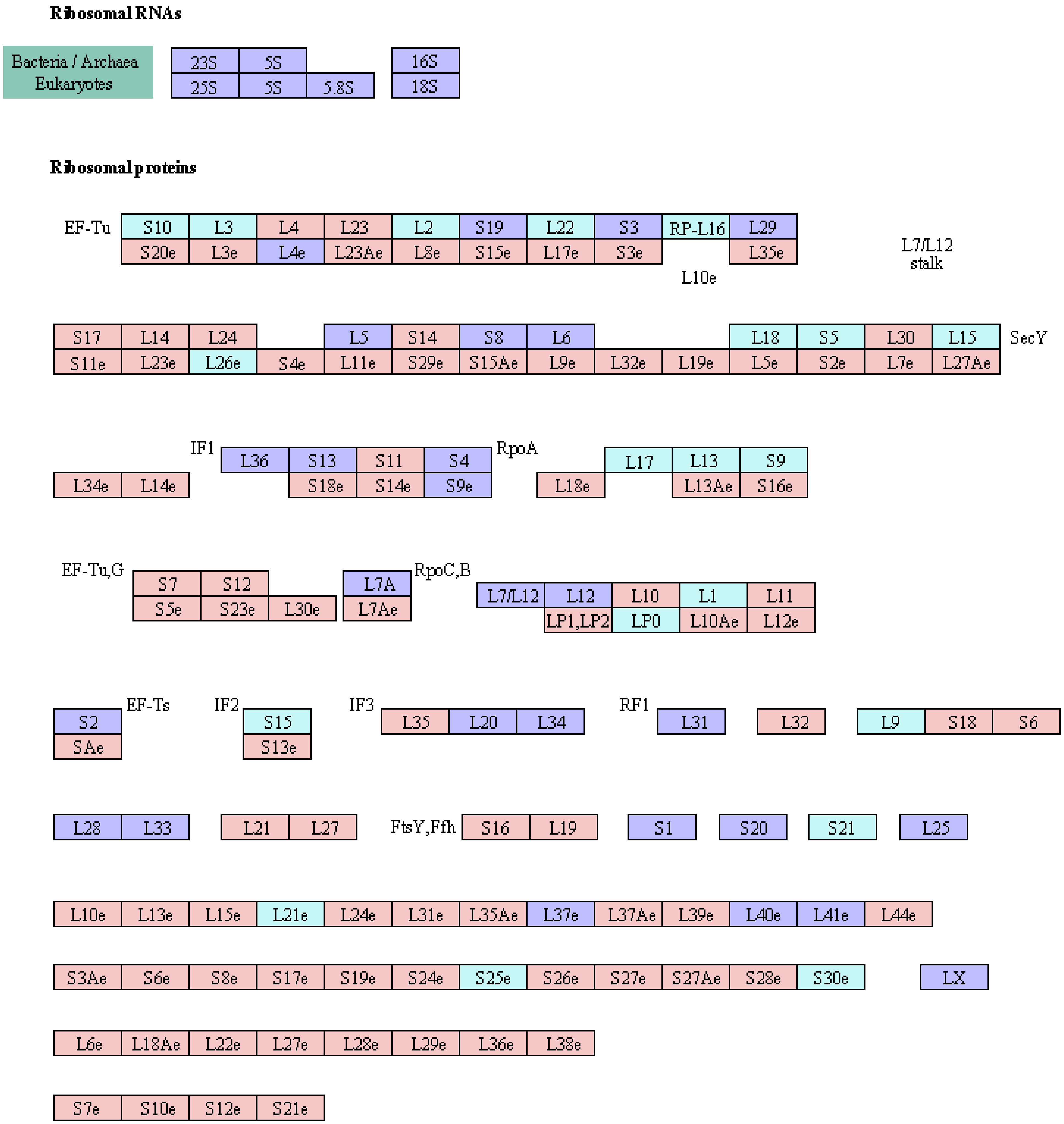


03010 7/29/13
(c) Kanehisa Laboratories

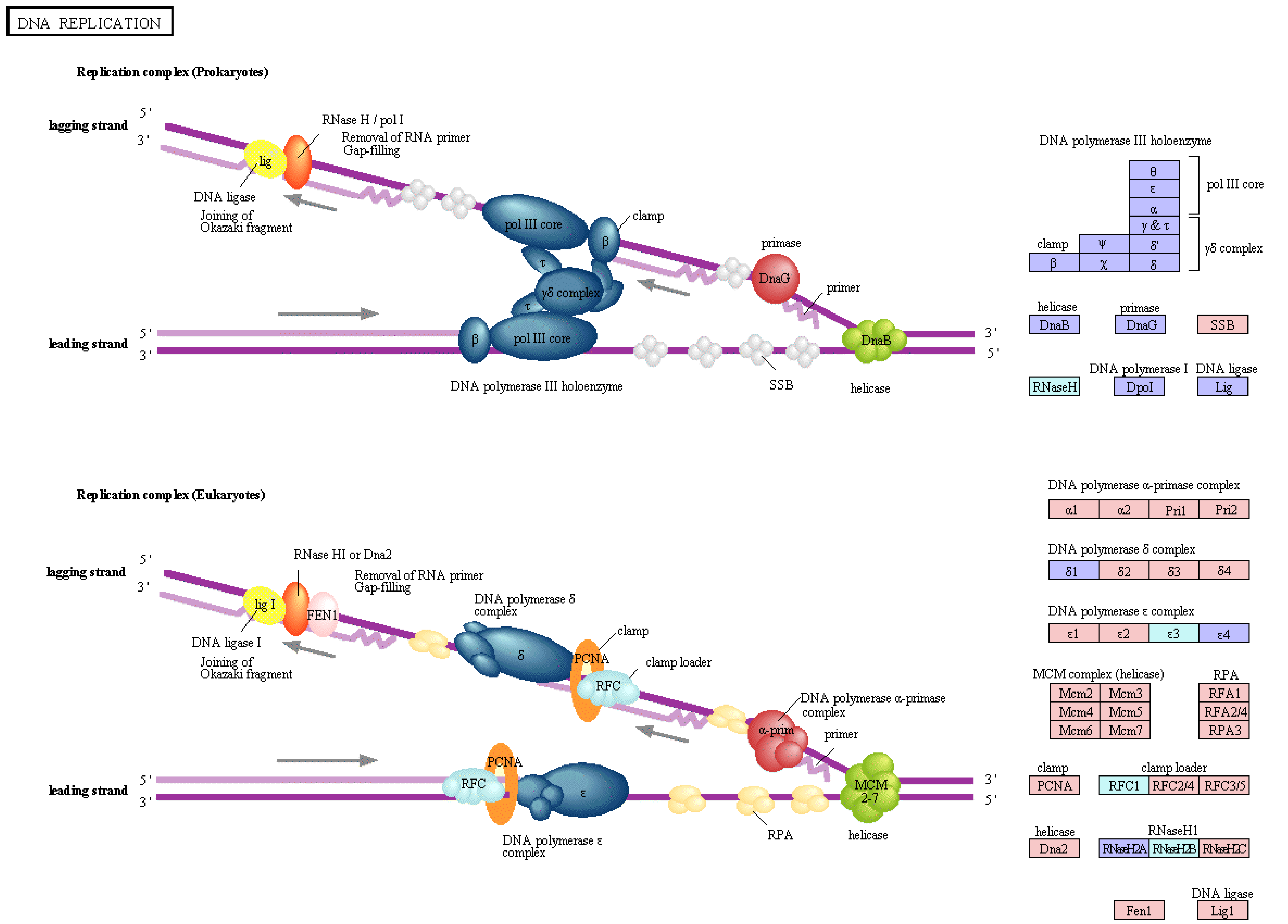


03030 10/20/10

(c) Kanehisa Laboratories

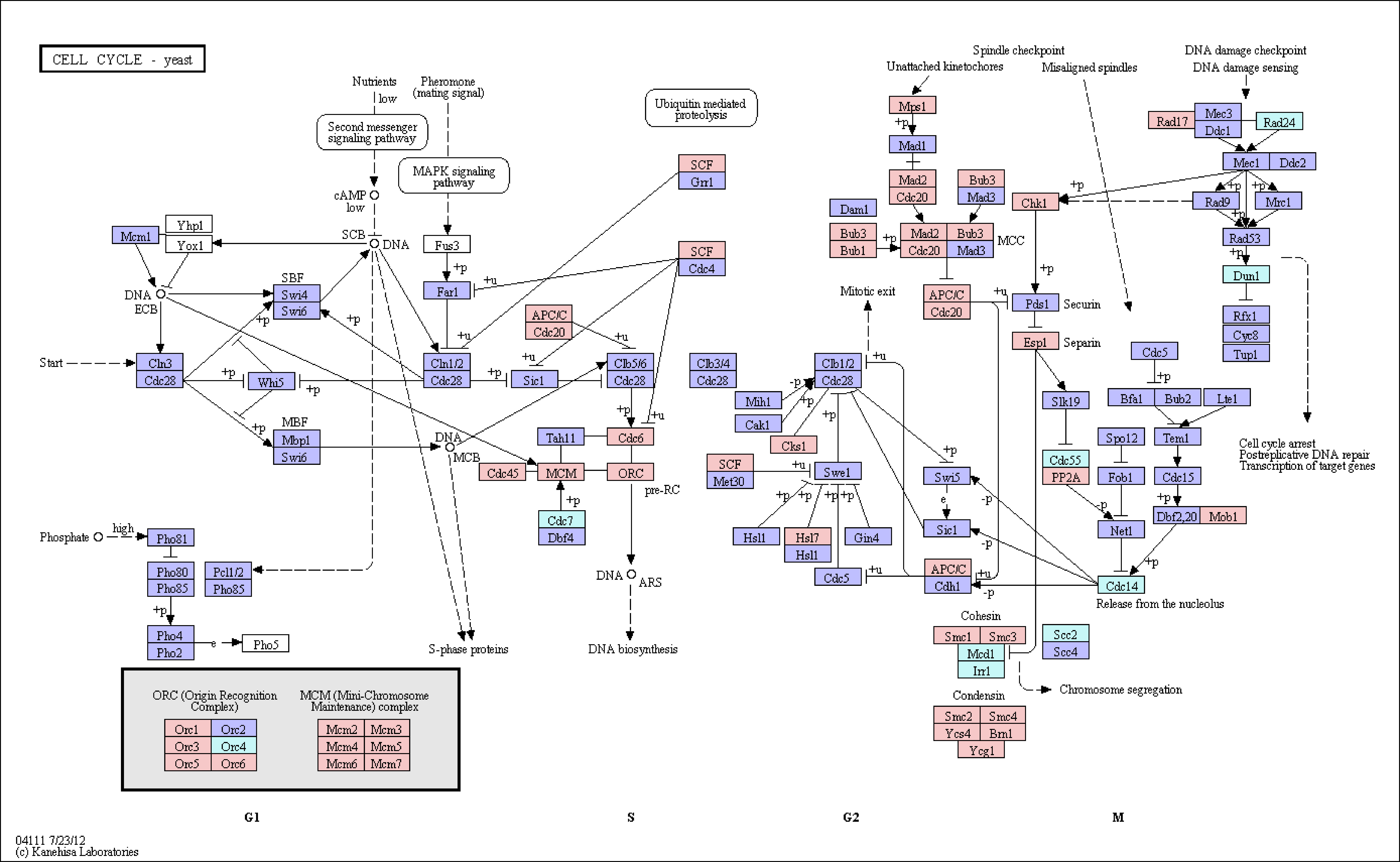
CELL CYCLE - yeast
Nutrients
low
Pheromone (mating signal)
Second messenger signaling pathway
MAPK signaling pathway
Ubiquitin mediated proteolysis
cAMP
low
SCB
DNA
Mcm1
Yhp1
Yox1
Fus3
Far1
SCF
Grr1
SCF
Cdc4
DNA
ECB
SBF
Swi4
Swi6
Start
Cln3
Cdc28
Whi5
Cln1/2
Cdc28
APC/C
Cdc20
Sic1
Clb5/6
Cdc28
Clb3/4
Cdc28
MBF
Mbp1
Swi6
DNA
MCB
Tah11
Cdc6
Cdc45
MCM
ORC
pre-RC
Cdc7
Dbf4
DNA
ARS
Phosphate
high
Pho81
Pho80
Pho85
Pcl1/2
Pho85
Pho4
Pho2
Pho5
S-phase proteins
DNA biosynthesis
ORC (Origin Recognition Complex)
Orc1
Orc2
Orc3
Orc4
Orc5
Orc6
MCM (Mini-Chromosome Maintenance) complex
Mcm2
Mcm3
Mcm4
Mcm5
Mcm6
Mcm7
Spindle checkpoint
Unattached kinetochores
Misaligned spindles
Mps1
Mad1
Mad2
Cdc20
Bub3
Mad3
Dam1
Bub3
Bub1
Mad2
Bub3
Cdc20
Mad3
MCC
APC/C
Cdc20
Mitotic exit
Clb1/2
Cdc28
Mih1
Cak1
Cks1
SCF
Met30
Swe1
Hsl1
Hsl7
Hsl1
Gin4
Cdc5
APC/C
Cdh1
Swi5
Sic1
Chk1
Pds1
Securin
Esp1
Separin
Slk19
Cdc55
PP2A
Cdc5
Bfa1
Bub2
Lte1
Spo12
Fob1
Net1
Tem1
Cdc15
Dbf2,20
Mob1
Cdc14
Release from the nucleolus
Cohesin
Smc1
Smc3
Mcd1
Irr1
Scc2
Scc4
Chromosome segregation
Condensin
Smc2
Smc4
Ycs4
Brn1
Ycg1
DNA damage checkpoint
DNA damage sensing
Rad17
Mec3
Ddc1
Rad24
Mec1
Ddc2
Rad9
Mrc1
Rad53
Dun1
Rfx1
Crt1
Tup1
Cell cycle arrest
Postreplicative DNA repair
Transcription of target genes
G1
S
G2
M
04111 7/23/12
(c) Kanehisa Laboratories

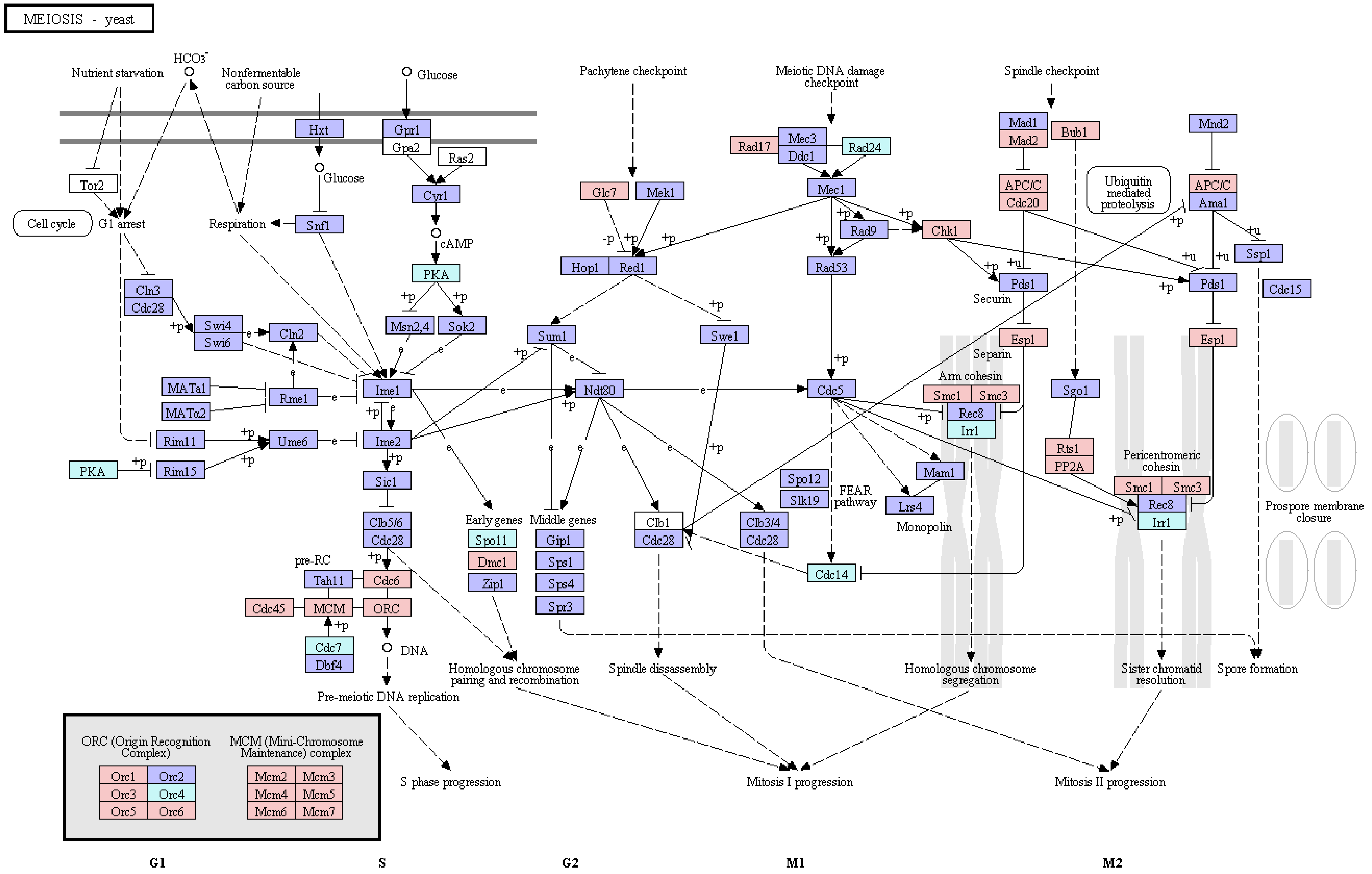


04113 5/13/14
(c) Kanehisa Laboratories

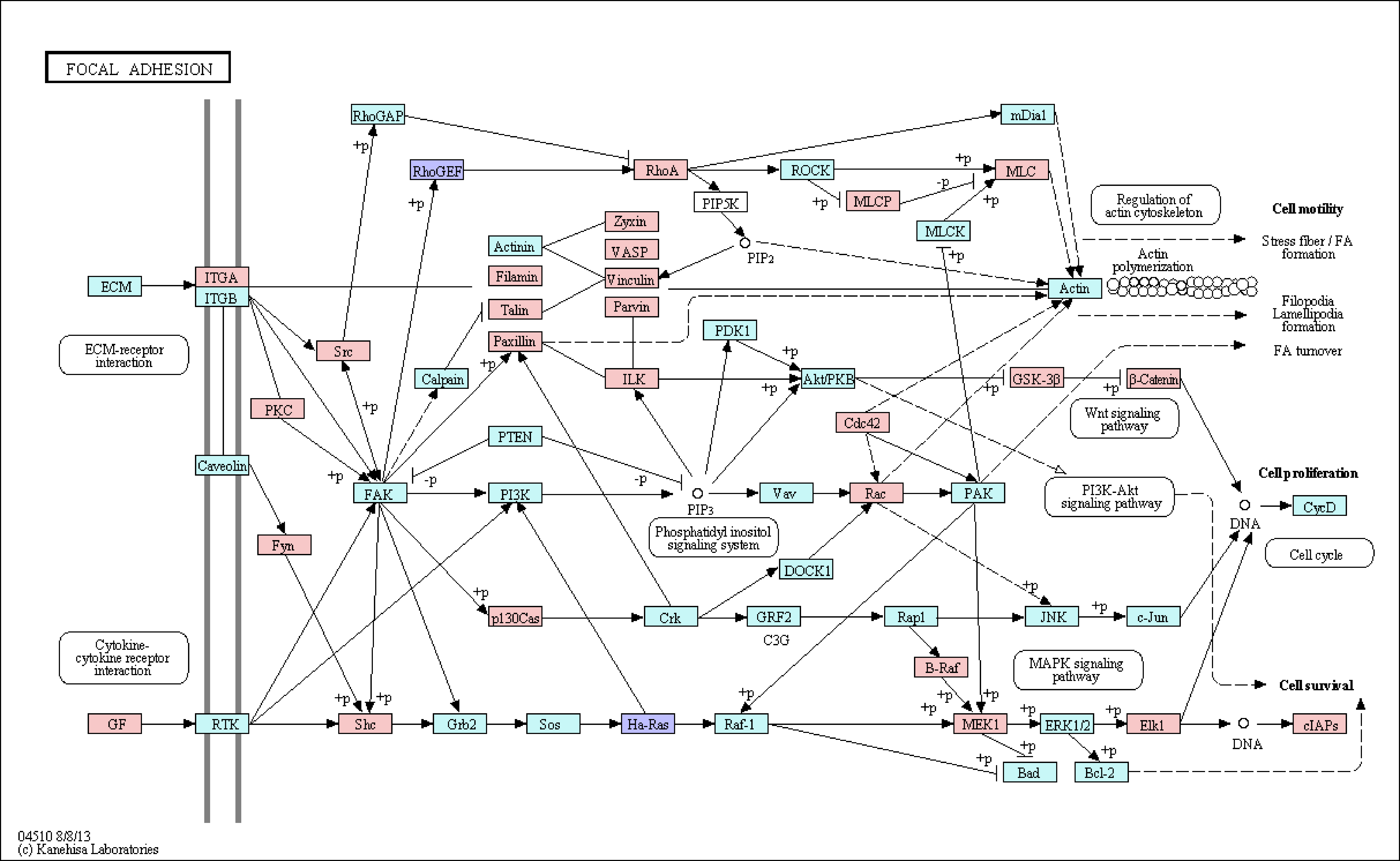
FOCAL ADHESION
ECM
ITGA
ITGB
Caveolin
RTK
GF
ECM-receptor interaction
Cytokine-cytokine receptor interaction
RhoGAP
RhoGEF
RhoA
PIP5K
ROCK
MLCP
MLC
mDia1
MLCK
Zyxin
VASP
Actinin
Filamin
Vinculin
Talin
Parvin
Paxillin
PIP2
Src
PKC
Calpain
ILK
PDK1
Akt/PKB
GSK-3β
β-Catenin
Cdc42
PTEN
FAK
PI3K
PIP3
Vav
Rac
PAK
Phosphatidyl inositol signaling system
DOCK1
Fyn
p130Cas
Crk
GRF2
C3G
Rap1
JNK
c-Jun
B-Raf
Shc
Grb2
Sos
Ha-Ras
Raf-1
MEK1
ERK1/2
Elk1
Bad
Bcl-2
DNA
cIAPs
CycD
Actin
Actin polymerization
Regulation of actin cytoskeleton
Cell motility
Stress fiber / FA formation
Filopodia Lamellipodia formation
FA turnover
Wnt signaling pathway
PI3K-Akt signaling pathway
Cell proliferation
Cell cycle
MAPK signaling pathway
Cell survival
+p
-p
04510 8/8/13
(c) Kanehisa Laboratories

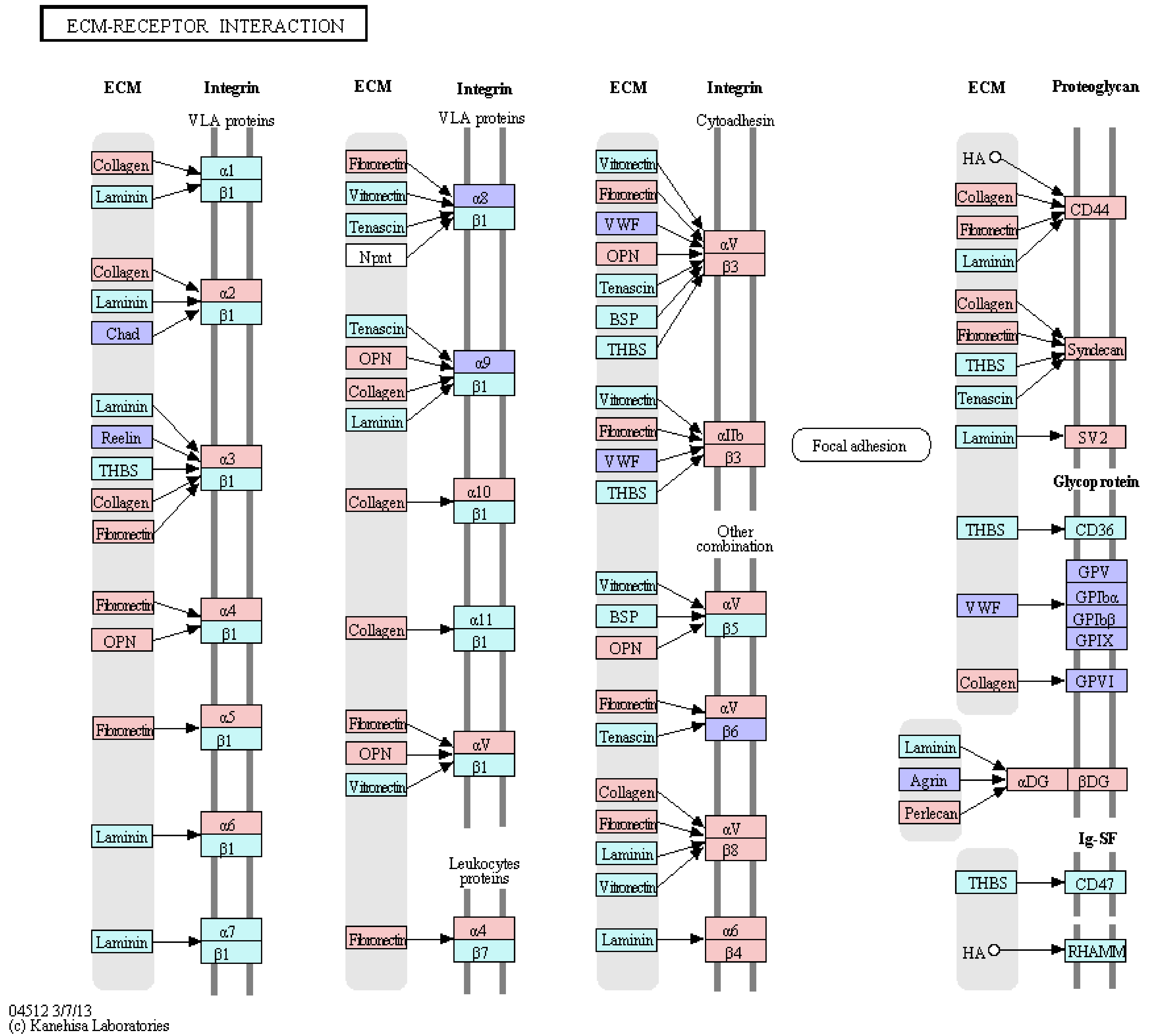
ECM-RECEPTOR INTERACTION
ECM
Integrin
VLA proteins
Collagen
Laminin
α1
β1
Collagen
Laminin
Chad
α2
β1
Laminin
Reelin
THBS
Collagen
Fibronectin
α3
β1
Fibronectin
OPN
α4
β1
Fibronectin
α5
β1
Laminin
α6
β1
Laminin
α7
β1
ECM
Integrin
VLA proteins
Fibronectin
Vitronectin
Tenascin
Npnt
α8
β1
Tenascin
OPN
Collagen
Laminin
α9
β1
Collagen
α10
β1
Collagen
α11
β1
Fibronectin
OPN
Vitronectin
αV
β1
Leukocytes proteins
Fibronectin
α4
β7
ECM
Integrin
Cytoadhesin
Vitronectin
Fibronectin
VWF
OPN
Tenascin
BSP
THBS
αV
β3
Vitronectin
Fibronectin
VWF
THBS
αIIb
β3
Other combination
Vitronectin
BSP
OPN
αV
β5
Fibronectin
Tenascin
αV
β6
Collagen
Fibronectin
Laminin
Vitronectin
αV
β8
Laminin
α6
β4
Focal adhesion
ECM
Proteoglycan
HA
Collagen
Fibronectin
Laminin
CD44
Collagen
Fibronectin
THBS
Tenascin
Syndecan
Laminin
SV2
Glycoprotein
THBS
CD36
VWF
GPV
GPIbα
GPIbβ
GPIX
Collagen
GPVI
Laminin
Agrin
Perlecan
αDG
βDG
Ig-SF
THBS
CD47
HA
RHAMM
04512 3/7/13
(c) Kanehisa Laboratories

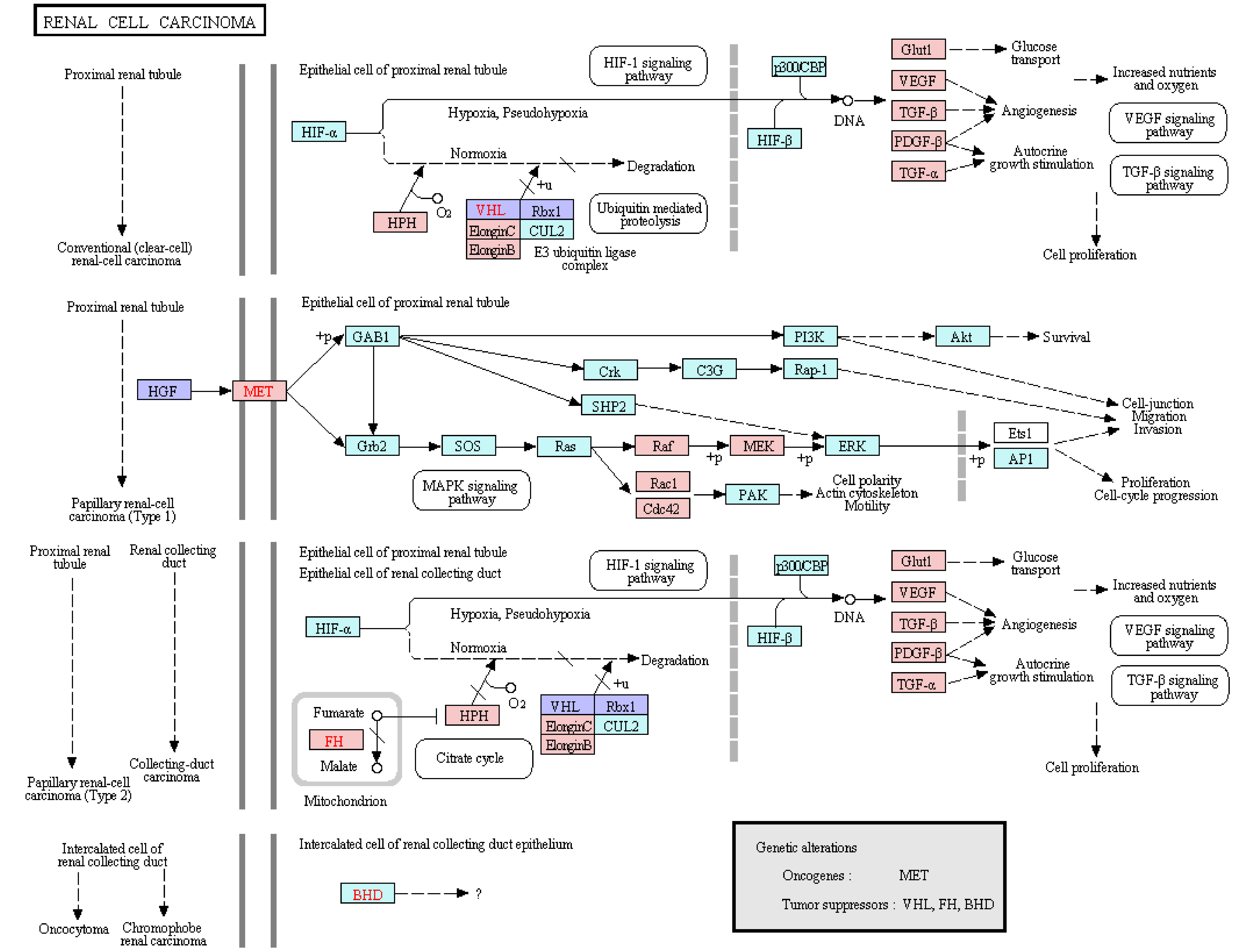


05211 7/24/13
(c) Kanehisa Laboratories
